# Extraction, Purification and Identification of Bovine Lung Peptides and Its Antioxidant Effects on H_2_O_2_-Induced HepG2 Cells and Mice with Alcoholic Liver Injury

**DOI:** 10.3390/antiox14111314

**Published:** 2025-10-31

**Authors:** Xingyu Xiao, Xunming Zhang, Yi Li, Tong Su, Shuo Zheng, Jiayuan Fang, Qinchuan Lv, Dacheng Wang, Linlin Hao

**Affiliations:** College of Animal Science, Jilin University, Changchun 130062, China; xyxiao23@mails.jlu.edu.cn (X.X.); xunming22@mails.jlu.edu.cn (X.Z.); yil22@mails.jlu.edu.cn (Y.L.); sutong9921@mails.jlu.edu.cn (T.S.); zhengshuo22@mails.jlu.edu.cn (S.Z.); fangjy23@mails.jlu.edu.cn (J.F.); lvqc9921@mails.jlu.edu.cn (Q.L.)

**Keywords:** antioxidant peptides, bovine lung, alcoholic liver injury, Keap1, molecular docking

## Abstract

In this study, we constructed an extraction process for bovine lung peptide-1 (BLP-1) derived from bovine lung tissue utilizing single-factor optimization combined with response surface methodology. We systematically analyzed its antioxidant activity, biological safety, and therapeutic mechanisms against alcoholic liver disease (ALD). In vitro experiments demonstrated that BLP-1 exhibits excellent scavenging activity against various free radicals, while exhibiting no significant cytotoxicity or hemolytic activity. In a model of H_2_O_2_-induced oxidative damage in HepG2 cells, BLP-1 significantly alleviated oxidative stress injury by upregulating the activities of intracellular antioxidant enzymes. Animal experiments further confirmed that BLP-1 significantly reduced serum levels of transaminase, inhibited the release of inflammatory factors, enhanced antioxidant enzyme activity, and ameliorated lipid peroxidation and pathological injury in ALD mice. By combining liquid chromatography-tandem mass spectrometry (LC-MS/MS) with bioinformatics, we screened 12 novel antioxidant peptides. Among these, the binding energies of GP9, FG6, and WG6 to Keap1 were −10.2, −9.7, and −8.7 kcal/mol, respectively, indicating their potential to modulate the antioxidant defense system through competitive inhibition of Keap1-Nrf2 interactions. This study provides a novel approach for the high-value utilization of bovine lung and the treatment of ALD, as well as a new source for the extraction of natural antioxidant peptides.

## 1. Introduction

Alcohol, as a highly addictive psychotropic substance, has constituted a major public health crisis due to its global abuse [1,2]. Epidemiological data indicate that alcohol-related diseases rank seventh in the global disease burden, directly accounting for 3.8% of deaths and 4.6% of disability-adjusted life years lost [3,4]. Among the many alcohol-related diseases, alcoholic liver disease (ALD) has emerged as a prominent concern due to its high incidence [5]. Although abstinence from alcohol is established as the cornerstone strategy in the treatment of ALD, practical challenges, such as insufficient patient compliance and irreversible late-stage pathological damage, underscore the urgent need for the development of new treatment strategies [6].

The liver, as the primary organ responsible for alcohol metabolism, is tasked with approximately 90% of ethanol conversion [7]. The key metabolic enzyme, cytochrome P450 2E1 (CYP2E1), generates a significant amount of reactive oxygen species (ROS) during its catalytic activity, leading to an imbalance in the dynamic equilibrium of the body’s oxidation-antioxidation system [8,9]. This state of oxidative stress not only directly induces lipid peroxidation and DNA damage but also activates Kupffer cells, which release pro-inflammatory factors such as tumor necrosis factor-α (TNF-α) and Interleukin 6 (IL-6), thereby establishing a vicious cycle of “oxidative stress-inflammatory response” [10,11]. Silymarin, a widely recognized liver protectant, has been shown to ameliorate ALD by scavenging reactive oxygen species and restoring antioxidant enzyme activity. However, its clinical application is limited by low bioavailability and potential biotoxicity [12,13,14]. Consequently, there has been significant interest in natural antioxidants with high antioxidant activity and biocompatibility, particularly antioxidant peptides, for the treatment of ALD.

Research indicates that antioxidant peptides not only directly neutralize ROS but also enhance the expression of antioxidant enzymes through the activation of the Keap1-Nrf2 pathway, while simultaneously inhibiting the NF-κB-mediated inflammatory cascade [15,16]. In recent years, significant advancements have been made in the study of antioxidant peptides derived from marine organisms and plants [17,18]. The potential of antioxidant peptides sourced from animals, particularly those from mammalian organs, remains largely unexplored. As a slaughter by-product, bovine lung is rich in collagen and contains potential active peptide segments [19,20]. Its high protein content and underutilized status make it an ideal source of functional peptides [21,22]. Previous studies have confirmed that bovine lung hydrolysate possesses anti-inflammatory activity [23]. However, its antioxidant properties and applications in ALD have not been systematically explored.

In recent years, the application of liquid chromatography-tandem mass spectrometry (LC-MS/MS) technology has significantly accelerated the rapid development of peptidomics research. This technique facilitates the swift characterization of peptide information in enzymatic hydrolysates and, when combined with relevant databases such as PeptideRanker and BIOPEP, enables the activity screening of the vast array of peptides identified in peptidomics. This advancement effectively addresses the efficiency bottleneck associated with traditional separation and purification methods. Currently, this technology is widely employed in the identification of antioxidant peptides, anti-inflammatory peptides, and α-glucosidase inhibitory peptides [24,25]. Additionally, the maturation of molecular docking technology has introduced a precise virtual research tool for elucidating the mechanisms of peptide action. By simulating the binding modes of peptide segments with target proteins (such as Keap1), researchers can identify high-potency active peptides based on binding energy and interaction site analysis, thereby providing deeper insights into the mechanisms through which they exert their biological activity.

This study successfully isolated a low molecular weight (<3 kDa) bioactive peptide fraction, designated as bovine lung peptide-1 (BLP-1), from bovine lung, demonstrating significant antioxidant activity. In the oxidative damage HepG2 cell model, BLP-1 markedly alleviated cellular oxidative damage by enhancing antioxidant enzyme activity and reducing malondialdehyde (MDA) levels. In the ALD mouse model, BLP-1 exhibited multi-dimensional protective effects by decreasing the release of transaminases and inflammatory factors while upregulating antioxidant enzyme activity in liver tissue. Additionally, twelve novel antioxidant peptide sequences were identified from BLP-1 using peptidomics. Molecular docking analyses indicated that these peptide sequences could exert antioxidant activity via the Keap1-Nrf2 signaling pathway. Therefore, this study provides a scientific paradigm for the high-value utilization of animal by-products and also offers a new approach for the treatment of ALD.

## 2. Materials and Methods

### 2.1. Materials and Reagents

The bovine lung samples used in this experiment were purchased from local slaughterhouses and were preserved at −80 °C for future use. According to the Laboratory Animal Ethics Committee of Jilin University, ethical approval is not required for the use of commercially procured tissues. Compound protease (120 U/mg), alkaline protease (200 U/mg), flavor protease (20 U/mg), papain (800 U/mg), and neutral protease (100 U/mg) were procured from Shanghai Yuanye Biotechnology Co., Ltd. (Shanghai, China). The cell viability detection kit (CCK8) was sourced from MEC Company (Shanghai, China). Detection kits for DPPH, ABTS, superoxide anion, and hydroxyl radical scavenging rates, as well as biochemical indicators superoxide pismutase (SOD), glutathione peroxidase (GSH-Px), catalase (CAT), MDA, aspartate aminotransferase (AST), alanine aminotransferase (ALT), were obtained from Nanjing Jiancheng Bioengineering Research Institut (Nanjing, China). The IL-6 and TNF-α ELISA detection kits were purchased from Shanghai Qifa Experimental Reagent Co., Ltd. (Shanghai, China). Hematoxylin and eosin solutions were acquired from Solarbio Technology Ltd. (Beijing, China). The target peptides were synthesized using the solid-phase synthesis method by SynPeptide Co., Ltd. (Nanjing, China). HepG2 cells were maintained in our laboratory. All other chemicals and reagents were of chromatographic or analytical grade.

### 2.2. Preparation of BLP

#### 2.2.1. Pretreatment of Bovine Lungs

Fresh bovine lung tissue was retrieved from −80 °C and thawed. The tissue was then thoroughly rinsed with distilled water to eliminate residual impurities. Subsequently, it was cut into small pieces and subjected to fine homogenization.

#### 2.2.2. Screening of Proteases

Lung tissue homogenate was mixed with distilled water at a solid–liquid ratio of 10% (*w*/*v*) and shaken thoroughly. This mixture was divided into five groups of parallel samples for differential enzymatic hydrolysis experiments. Each group received a specific protease: compound protease, alkaline protease, flavor protease, papain protease, and neutral protease, each with an enzyme activity of 2000 U/g. The enzymatic hydrolysis was conducted at the respective optimal temperatures and pH conditions for a duration of 3 h. After the enzymatic hydrolysis, the product was centrifuged (4 °C, 4000 rpm, 10 min) using a SORVALL Biofuge Stratos high-speed refrigerated centrifuge (Thermo Fisher, Waltham, MA, USA) equipped with rotor model #3335, and the supernatant was collected. The supernatant was heated in boiling water for 5 min to inactivate the protease, resulting in the acquisition of the BLP crude extract. The DPPH and ABTS free radical scavenging rates were employed as evaluation indicators to assess the antioxidant activity of BLP.

### 2.3. Optimization of BLP Extraction Conditions

#### 2.3.1. Single-Factor Experiment

Based on the results of protease screening presented in Section 2.2, the effects of hydrolysis conditions on the DPPH and ABTS radical scavenging rates were investigated using papain as the hydrolytic enzyme through single-factor experiments. The following single-factor variables were established for the experiments: enzyme concentration (1000, 2000, 3000, 4000 U/g), hydrolysis time (2, 3, 4, 5, 6 h), material–liquid ratio (5%, 10%, 15%, 20%, 25%), enzyme hydrolysis temperature (40 °C, 45 °C, 50 °C, 55 °C, 60 °C), and pH value (5, 6, 7, 8, 9). During the optimization of the enzymatic hydrolysis conditions, only one variable was altered at a time while keeping all other conditions constant.

#### 2.3.2. Design of Response Surface Methodology (RSM)

Based on the results of the single-factor experiments, enzyme concentration (A), pH (B), and enzymatic hydrolysis time (C) were selected as independent variables, with the DPPH and ABTS clearance rates serving as response variables. The Box–Behnken design was employed to further optimize the extraction parameters. The factors and levels of the experimental design are presented in Supplementary Table S1.

### 2.4. Isolation and Purification of BLP

#### 2.4.1. Ultrafiltration

The BLP obtained after optimizing the extraction process was separated and purified using ultrafiltration membranes with specifications of 3 kDa and 10 kDa (Millipore, Burlington, MA, USA), resulting in three fractions with molecular weights of >10 kDa, 3–10 kDa, and <3 kDa, respectively. After pre-freezing all fraction at −80 °C, they were lyophilized using a freeze dryer (Xinzhi, Ningbo, China) at a pressure of 1 Pa and a temperature of −45 °C for a duration of 24 h. The resulting lyophilized powder was collected and stored at −80 °C for subsequent experiments.

#### 2.4.2. Gel Filtration Chromatography

Dissolve the components with a molecular weight below 3 kDa, as described in Section 2.4.1, in water to prepare a 5 mL solution at a concentration of 20 mg/mL. Subsequently, purify this solution further using a Sephadex G-25 gel chromatography column (Cytiva, Marlborough, MA, USA) with dimensions of 1.0 × 40 cm. Elution was conducted with ultrapure water at a flow rate of 1 mL/min. Fractions exhibiting absorption peaks at 220 nm were collected and lyophilized for subsequent testing of their radical scavenging activity.

### 2.5. Molecular Weight Determination

The molecular weight (Mw) distribution of the BLP-1 was determined using a Waters 2695 high-performance liquid chromatography system equipped (Waters, Milford, MA, USA) with a 300 mm × 7.8 mm TSKgel 2000SWxl column (Tosoh Bioscience, Tokyo, Japan). The mobile phase for the measurement comprised acetonitrile/water/trifluoroacetic acid (40:60:0.1, *v*/*v*), with a flow rate of 0.5 mL/min, a column temperature of 30 °C, and a detection wavelength of 220 nm. Additionally, the following standards were employed to construct the molecular weight calibration curve: cytochrome (MW 12384), aprotinin (MW 6500), bacitracin (MW 1422), Gly-Gly-Tyr-Arg (MW 451), and Gly-Gly-Gly (MW 189).

### 2.6. Biosafety Assessment

#### 2.6.1. Cytotoxicity Assay

The cytotoxicity assay was conducted according to the methodology outlined in previous studies with minor modifications [26]. Specifically, HepG2 cells (1 × 10^4^ cells/well) were cultured for 24 h and subsequently treated with varying concentrations of BLP-1 (100, 200, 400, 600, 800, 1000 μg/mL). After 24 h of incubation, the medium was discarded, and the cells were washed with PBS. Finally, cell viability was assessed using the CCK8 kit.

#### 2.6.2. Hemolysis Test

Collected mouse blood cells were washed with phosphate-buffered saline (PBS) and resuspended to prepare a red blood cell suspension with a final concentration of 1% (*v*/*v*). The red blood cell suspension was mixed with an equal volume of BLP-1 at various concentrations (1, 2, 4, 8, 16 mg/mL) and incubated at 37 °C for 1 h. The incubated mixture was then centrifuged (4000 rpm, 3 min) using the SORVALL Biofuge Stratos high-speed refrigerated centrifuge to obtain the supernatant, and the absorbance was measured at 570 nm. In this experiment, PBS and 1% Triton-X-100 were used as the negative and positive controls, respectively.

### 2.7. Evaluation of Antioxidant Activity In Vitro

The in vitro antioxidant capacity of BLP-1 was evaluated by assessing its scavenging ability against DPPH, ABTS, hydroxyl radicals, and superoxide anions at various concentrations following the methodologies established in prior studies [27]. To provide a more intuitive comparison of the in vitro antioxidant capacity of BLP-1, glutathione (GSH) was utilized as the positive control.

### 2.8. Protective Effect of BLP-1 on HepG2 Cells Against Oxidative Stress

#### 2.8.1. Cell Experimental Design

Firstly, the cryopreserved HepG2 cells were thawed and cultured in DMEM medium under conditions of 5% CO_2_ at 37 °C until reaching the appropriate density. Subsequently, the cells were prepared as a suspension at a concentration of 1 × 10^5^ cells/mL and subjected to different group culture for 24 h, which included the control group, damage group (750 μmol/L H_2_O_2_), and treatment group with different doses of BLP-1 (100, 200, 400, 600 μg/mL). Next, an appropriate concentration of H_2_O_2_ was introduced to induce oxidative damage, followed by a 2 h incubation period. Finally, the oxidative damage-related indicators (SOD, CAT, GSH-Px, and MDA) were measured.

#### 2.8.2. Selection of H_2_O_2_ Concentration

To establish a model of oxidative stress injury and to screen for optimal injury conditions, this study treated HepG2 cells with various concentrations of H_2_O_2_ (650, 700, 750, 800, 850 μmol/L) to determine the concentration that induces half cell death, which was subsequently used for further experiments.

#### 2.8.3. The Effect of BLP-1 on Cell Viability

To evaluate the protective effect of BLP-1 on oxidatively damaged HepG2 cells, we incubated the cells with DMEM supplemented with varying concentrations of BLP-1(100, 200, 400, 600, 800, 1000 μg/mL). Subsequently, the cells were co-incubated with the H_2_O_2_ concentrations specified in Section 2.8.2. The cell viability of different groups was measured using the CCK8 assay.

#### 2.8.4. Determination of Antioxidant Enzyme Activity and MDA Level

After incubating HepG2 cells in DMEM at concentrations of 100, 200, 400, and 600 μg/mL, and subsequently co-incubating with the H_2_O_2_ concentrations identified in Section 2.8.2, the activities of SOD, CAT, GSH-Px, and the level of MDA were measured using an assay kit.

### 2.9. Protective Effect of BLP-1 on Mice with ALD

#### 2.9.1. Animal Experiment

Forty male Kunming mice, aged six weeks, were purchased from Liaoning Changsheng Biotechnology Co., Ltd. (Benxi, China) and housed for 15 days under controlled conditions of 22 ± 0.5 °C temperature, 55 ± 5% humidity, and a 12 h light–dark cycle. During this housing period, the mice had unrestricted access to a standard laboratory diet and water. All experiments were conducted in accordance with ethical protocols and standards for laboratory animals and were supervised and approved by the Laboratory Animal Ethics Committee of Jilin University (Approval No. SY202412055; Approval date: 7 January 2025).

The animal experimental design and schedule are illustrated in Figure 5A. The groups included a control group (0.9% NaCl), a model group (53% ethanol), and three treatment groups with low, medium, and high doses. Based on previous studies concerning the treatment of ALD with bovine bone collagen peptide, the treatment groups were administered BLP-1 at concentrations of 300 mg/mL, 600 mg/mL, and 1200 mg/mL, corresponding to low, medium, and high dose groups, respectively [28]. All mice were gavaged in a volume of 0.12 mL/10 g. The various doses were prepared by dissolving different amounts of BLP-1 in ethanol at a volume fraction of 53%. During the experiment, each group was gavaged for eight consecutive days, followed by an additional gavage on the eighth day after a one-hour interval. Except for the control group, which received 0.9% NaCl, all other groups were administered 53% ethanol by volume. After the final gavage, the mice were fasted without food or water. Twelve hours later, the mice were weighed, and blood was collected via eyeball removal. The upper serum layer was centrifuged and stored in a refrigerator at −80 °C for future analysis. Subsequently, following euthanasia, the livers were removed, rinsed with saline, weighed, and the liver index was calculated. Portions of the livers were fixed in 4% paraformaldehyde, while others were stored in −80 °C.

#### 2.9.2. Biochemical Testing and ELISA Assay

Fresh liver was homogenized with PBS (1:9, *v*/*v*) and then subjected to centrifugation to obtain the supernatant. The levels of antioxidant enzymes, including SOD, CAT, GSH-Px, and MDA, were measured in the liver using commercial kits. Furthermore, the activities of AST and ALT, as well as the levels of inflammatory factors TNF-α and IL-6, were evaluated in serum through biochemical assays and ELISA kits.

#### 2.9.3. Histopathological Analysis

Liver tissue fixed with 4% paraformaldehyde was dehydrated and embedded in paraffin. The tissue was then sectioned into slices of 5 μm thickness and stained with hematoxylin and eosin (H&E). Finally, the sections were photographed at magnifications of ×200 and ×400 to observe histopathological changes.

### 2.10. Identification of Antioxidant Peptide Sequences in BLP-1

#### 2.10.1. LC-MS/MS Identification

To further elucidate the sequence composition of BLP-1, we conducted an LC-MS/MS analysis using the VANQUISH NEO nanoliter liquid chromatography system (Thermo Fisher, Waltham, MA, USA) to identify the peptide sequences present in BLP-1. Prior to detection, BLP-1 should be dissolved and desalted, followed by lyophilization and reconstitution in a 0.1% formic acid solution. The supernatant is then collected by centrifugation for sample detection. During the detection process, mobile phase A consisted of 0.1% formic acid, while mobile phase B comprised 80% acetonitrile containing 0.1% formic acid. Upon completion of the assay, information regarding the peptide sequences in BLP-1 was obtained by comparing them with the protein sequence of Bos taurus available in UniProt.

#### 2.10.2. Antioxidant Peptide Sequence Screening

Utilize the online tool PeptideRanker (http://distilldeep.ucd.ie/PeptideRanker/, accessed on 12 July 2025) to predict the probability of biological activity for peptides, setting the screening threshold at 0.95. Additionally, employ the BIOPEP (https://biochemia.uwm.edu.pl/biopep/start_biopep.php, accessed on 12 July 2025) to assess the bioactivity of the peptide sequences obtained in the previous step, thereby identifying the antioxidant peptides present in BLP-1.

#### 2.10.3. Prediction of Antioxidant Peptide Properties

The screened antioxidant peptide sequences were analyzed using the APD3 database (https://aps.unmc.edu/AP/, accessed on 15 July 2025) to predict their charge, relative molecular weight, and GRAVY. ToxinPred (https://webs.iiitd.edu.in/raghava/toxinpred/, accessed on 15 July 2025) was utilized to assess peptide cytotoxicity.

### 2.11. Molecular Docking

#### 2.11.1. Docking with DPPH and ABTS

The antioxidant peptides obtained from screening were subjected to molecular docking with DPPH and ABTS to evaluate their antioxidant activity, following the method described by Liu et al. The structures of the DPPH radical (CID: 2735032) and the ABTS radical (CID: 5360881) were obtained from the PubChem database [29]. Prior to docking, peptide modeling was performed using the online platform (https://cloud.yinfotek.com, accessed on 24 July 2025). AutoDock Vina software 1.2.6 was employed for docking, utilizing DPPH and ABTS as ligands and the peptides as receptors. The docking results were subsequently analyzed using PyMOL 2.3.0 and Discovery Studio 2019.

#### 2.11.2. Docking with Keap1

The antioxidant peptide was docked with Keap1 to investigate its potential antioxidant effects through the Nrf2/Keap1 pathway. The crystal structure of Keap1 (PDB ID: 2FLU) was sourced from the PDB (https://www.rcsb.org/, accessed on 28 July 2025). The binding site of Keap1 is located at coordinates x:5, y:9, z:2 [30]. In the docking process, the antioxidant peptide acted as the ligand, while Keap1 served as the receptor, with a docking box size of 50 × 50 × 50 Å. The methods employed for docking and result analysis were consistent with those used for DPPH and ABTS docking.

### 2.12. Peptide Synthesis and Antioxidant Activity Assay

After synthesizing the obtained antioxidant peptides through solid-phase synthesis (purity > 95%), the IC50 values for free radical scavenging activity were determined using DPPH and ABTS as evaluation indicators. Additionally, the toxicity of the synthesized peptides was assessed using the cytotoxicity assay method outlined in Section 2.6.1.

### 2.13. Statistical Analysis

Each experiment was conducted a minimum of three times. All data are presented as Mean ± SD (Standard Deviation) and analyzed using one-way ANOVA. To identify specific group differences, Tukey’s multiple comparisons test was subsequently conducted. All statistical analyses were performed using GraphPad Prism 9.0 (San Diego, CA, USA). *p* < 0.05 was deemed statistically significant.

## 3. Results and Discussion

### 3.1. Optimal Protease Selection

**Figure 1:**
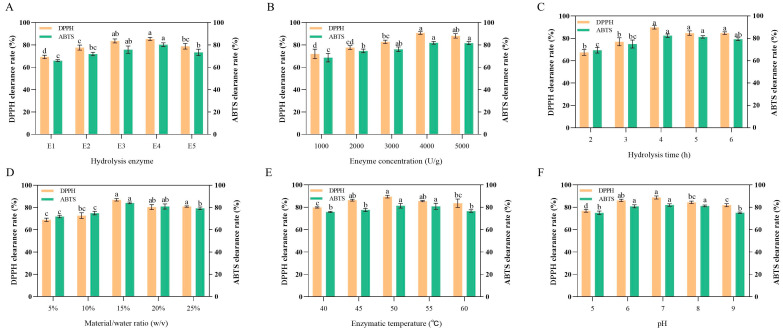
Protease Screening and Single-Factor Optimization of Hydrolysis for BLP. (**A**–**F**) demonstrate the effects of hydrolysis enzyme type, enzyme concentration, enzyme time, material/water ratio, enzymatic temperature, and pH on the DPPH and ABTS scavenging rates of BLP. E1–E5 represent compound protease, alkaline protein powder, flavor protease, papain, and neutral protease, respectively. All results were triplicates of the mean ± SD. Different lowercase letters represent significant difference (*p* < 0.05).

Proteases exhibit distinct substrate recognition and cleavage preferences during protein hydrolysis, attributable to specific differences in their enzymatic digestion sites [31]. This selectivity at the enzymatic site results in significant variations in peptide composition, molecular weight distribution, and amino acid sequences of the enzymatic products, ultimately leading to considerable differences in their antioxidant activity [18]. Consequently, the choice of appropriate proteases is a critical factor influencing the antioxidant activity of these enzymatic products. As illustrated in Figure 1A, five proteases were employed for the enzymatic hydrolysis of bovine lung, with the enzymatic products from flavor protease (E3) and papain (E4) exhibiting the most pronounced antioxidant activities when evaluated using DPPH and ABTS as assessment indices. Furthermore, the enzymatic products of flavor protease were noted to possess an irritating odor and slightly lower antioxidant activity compared to those derived from papain. Therefore, papain was selected for subsequent experiments. Similarly, the enzymatic hydrolysates of marine red algae (*Eucheuma cottonii*), sand eel (*Hypoptychus dybowskii*), small yellow croaker (*Pseudosciaena polyactis*), and venison treated with papain exhibited stronger antioxidant activity compared to those treated with other enzymes [32,33,34,35]. This is speculated to be due to papain’s superior protein hydrolytic capability [36].

### 3.2. Optimization of Single-Factor Experiment

After identifying papain as the optimal hydrolytic enzyme, we determined the optimal process conditions for BLP hydrolysis by screening key parameters during the enzymatic hydrolysis process. These parameters included enzyme concentration, hydrolysis time, material/water ratio, hydrolysis temperature, and pH value (Figure 1B–F). The optimal conditions were identified as follows: an enzyme concentration of 4000 U/g, a hydrolysis time of 4 h, a material/water ratio of 15%, a hydrolysis temperature of 50 °C, and a pH value of 7. Under these optimal conditions, the DPPH and ABTS radical scavenging capacities of BLP reached their peaks, demonstrating the best antioxidant activity.

From the perspective of various influencing parameters, when the enzyme concentration is below 4000 U/g, an increase in enzyme molecular density can promote substrate degradation, thereby enhancing antioxidant capacity. However, when the enzyme concentration exceeds this threshold, enzyme saturation in the system leads to excessive degradation of active components and resource wastage, resulting in a slight downward trend in activity [37,38]. In the early stages of hydrolysis, as the reaction time extends, the enzymatic hydrolysates gradually increase, and the activity exhibits a progressive upward trend, reaching its maximum after 4 h. Subsequently, high antioxidant activity peptides are further degraded into smaller molecular peptides or free amino acids, which leads to a reduction in activity [37]. When the material/water ratio is below 15%, the catalytic capacity of protease cannot be fully utilized due to insufficient and dispersed substrate, resulting in lower activity [39]. Conversely, when the ratio exceeds this percentage, the high viscosity of the system hinders enzyme diffusion, leading to a decrease in activity [40]. When the hydrolysis temperature is maintained within 45 °C, increased thermal motion of molecules promotes enzymatic reactions, gradually enhancing activity. However, when the temperature exceeds this range, enzyme denaturation and inactivation, along with changes in active sites, prevent effective binding with the substrate, causing a decline in activity [41]. When the pH value is at the optimal level of 7 for papain, its activity is maximized. Deviations from this optimal pH reduce the catalytic activity of the enzyme, thereby affecting its antioxidant efficacy [42].

### 3.3. Optimization of Hydrolysis Parameter by RSM

Based on the findings from single-factor experiments, enzyme concentration, pH, and hydrolysis time were selected for further optimization of the extraction conditions for BLP using RSM. Supplementary Table S2 presents the 17 experimental groups generated by Design-Expert, along with their corresponding DPPH and ABTS scavenging rates. The data were analyzed through multiple regression fitting using the corresponding software, resulting in regression model equations Y_1_ and Y_2_ for DPPH and ABTS, respectively.

Y_1_ = 87.27 − 0.7338A − 0.8650B − 0.5162C + 0.9325AB + 0.7150AC − 0.1225BC − 3.04A^2^ − 2.42B^2^ − 1.63C^2^

Y_2_ = 80.66 − 0.3850A − 0.7900B − 0.6650C + 0.4125AB + 0.7775AC − 0.1825BC − 2.96A^2^ − 1.91B^2^ − 2.65C^2^

Table 1 presents the variance analysis of the regression models for the DPPH and ABTS scavenging rates of BLP. The *p*-values for both models are ≤0.0001, indicating that these regression models are highly significant. The *p*-values for the lack of fit terms in the two models are 0.0965 and 0.3017, respectively, both exceeding 0.05. This demonstrates that the lack of fit terms is not significant. The R^2^ values for the DPPH and ABTS regression models are 0.9829 and 0.9734, respectively, with adjusted R^2^ values of 0.9563 and 0.9392. These results further validate the reliability of the models, which exhibit high degrees of fit, thereby allowing for their application in predicting and analyzing the enzymatic hydrolysis process of bovine lung.

**Figure 2:**
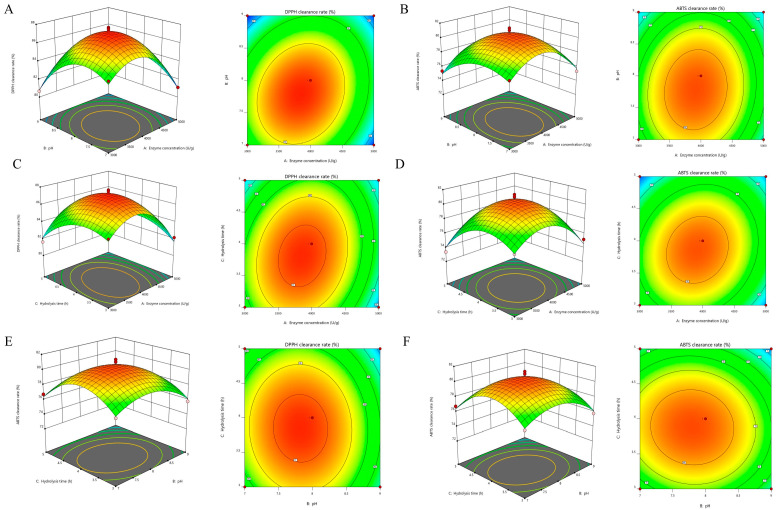
Response surface of DPPH and ABTS free radical scavenging rate for BLP. Images on the left represent three-dimensional response surface plots, whereas images on the right represent two-dimensional contour plots. (**A**,**B**) Effect of enzyme concentration and pH on the DPPH and ABTS free radical scavenging rate; (**C**,**D**) Effect of enzyme concentration and hydrolysis time on the DPPH and ABTS free radical scavenging rate; (**E**,**F**) Effect of pH and hydrolysis time on the DPPH and ABTS free radical scavenging rate. Red/orange hues indicate higher response values, while green/yellow hues indicate lower response values.

Furthermore, the interaction intensity among the factors can be effectively illustrated using three-dimensional response surfaces and contour plots. The steepness of the response surface is positively correlated with the degree of influence each factor exerts on the free radical scavenging rates of DPPH and ABTS. A steeper response surface indicates a greater influence of the factor on the response value, whereas a flatter surface suggests a lesser influence [43]. In the contour plots, a shape closer to an ellipse signifies a more significant interaction among the analyzed factors [44]. Consequently, the analysis presented in Figure 2 confirms that the effects of each factor on DPPH and ABTS clearance align with the findings from Table 1.

Under the DPPH model, the DPPH radical scavenging rate reached 86.12% at an enzyme concentration of 3730.11 U/g, a pH of 8.16, and an enzyme digestion time of 4.55 h. Additionally, under the ABTS model, the highest ABTS radical scavenging rate of 77.81% was achieved with an enzyme concentration of 3124.64 U/g, a pH of 7.26, and an enzyme digestion time of 3.21 h. To balance the optimal extraction conditions of both models while ensuring experimental operability, the extraction conditions were adjusted to an enzyme concentration of 3400 U/g, a pH of 7.70, and an enzyme digestion time of 3.9 h. Three replicate experiments were conducted to validate the optimized conditions, yielding an average DPPH radical scavenging rate of 89.17% and an ABTS radical scavenging rate of 82.78%, both of which showed minor discrepancies from the predicted values.

### 3.4. Separation and Purification of BLP

**Figure 3:**
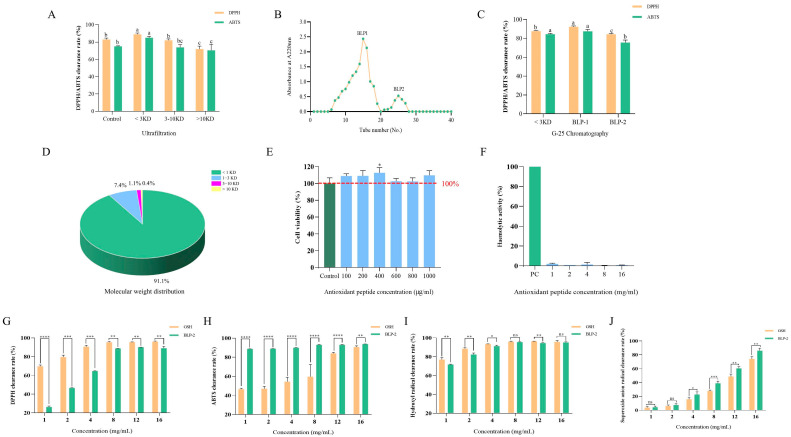
Purification and Physicochemical Characterization of BLP. (**A**) DPPH and ABTS free radical scavenging rate of BLP with three different molecular weights; (**B**) size exclusion chromatography of <3 kDa fraction; (**C**) DPPH and ABTS free radical scavenging rate of BLP-1 and BLP-2; (**D**) The molecular weight distribution of BLP; Cytotoxicity (**E**) and hemolytic activity (**F**) for BLP-1;DPPH (**G**), ABTS (**H**), hydroxyl radical (**I**), superoxide anion (**J**) scavenging rate for BLP-1. All results were triplicates of the mean ± SD. Different lowercase letters represent significant difference (*p* < 0.05), ns, *, **, ***, and **** represent no significance, *p* < 0.05, *p* < 0.01, *p* < 0.001, and *p* < 0.0001 respectively.

The antioxidant activity of enzymatic hydrolysates is closely related to their molecular weight. To investigate the differences in antioxidant activity of BLP with varying molecular weights, we employed ultrafiltration technology to separate BLP into three fractions based on molecular weight. Among these fractions, the one with a molecular weight of <3 kDa exhibited the highest antioxidant activity against DPPH and ABTS radicals, with values of 88.87 ± 1.46 and 84.95 ± 1.57, respectively (Figure 3A). This finding is consistent with the results reported by Wang and Yang et al., who demonstrated that enzymatic hydrolysates with a molecular weight of less than 3 kDa displayed the highest antioxidant activity [45,46]. Furthermore, the fraction of less than 3 kDa showed superior antioxidant activity compared to the non-ultrafiltered portion, indicating that ultrafiltration effectively enriches the antioxidant active substances in BLP. This aligns with previous conclusions that low molecular weight peptides exhibit stronger biological activities [47]. Consequently, the fraction of less than 3 kDa was selected for further research.

Subsequently, the <3 kDa fraction was further purified using gel filtration chromatography, resulting in the isolation of fractions BLP-1 and BLP-2 (Figure 3B). Fraction BLP-1 demonstrated a significantly stronger scavenging activity against DPPH and ABTS radicals in comparison to BLP-2 (Figure 3C). Analysis of the molecular weight distribution indicated that BLP-1 primarily comprised 91% peptides with a molecular weight below 1 kDa (Figure 3D). This finding suggests that the antioxidant peptides in BLP-1 are primarily low molecular weight peptides, which supports the notion that low molecular weight peptides exhibit greater antioxidant activity [48].

### 3.5. Biological Toxicity of BLP-1

The biosafety of a substance is a critical factor in evaluating its potential for medical and edible applications [49]. In this study, hemolytic activity and cytotoxicity were employed as indicators to assess the biosafety of BLP-1. When the concentration of BLP-1 ranged from 100 to 1000 μg/mL, it did not exhibit cytotoxicity compared to the control group and even promoted cell growth (Figure 3E). In the hemolysis test, even at a concentration of 16 mg/mL, DBP-1 did not induce hemolysis (Figure 3F). These results suggest that DBP-1, as an antioxidant peptide, demonstrates favorable biosafety and holds potential for further research and development.

### 3.6. Antioxidant Capacity of BLP-1 In Vitro

To further evaluate the in vitro antioxidant capacity of BLP-1, this study employed glutathione (GSH) as a positive control and assessed the antioxidant capacity of BLP-1 based on its scavenging ability against four common free radicals (Figure 3G–J). The results indicated that within the concentration range of 1–16 mg/mL, the scavenging capacity of BLP-1 against DPPH radicals, ABTS radicals, hydroxyl radicals, and superoxide anion radicals increased in a dose-dependent manner, reaching values of 90.16 ± 0.03%, 90.69 ± 1.19%, 95.15 ± 0.71%, and 74.12 ± 3.27%, respectively. With the exception of the superoxide anion, BLP-1 achieved a scavenging rate of 90% against the other three types of radicals. These findings suggest that BLP-1 possesses significant free radical scavenging ability, establishing it as a potent antioxidant with strong potential applications in antioxidant foods and cosmetics.

### 3.7. Cytoprotective Function of BLP-1 Against H_2_O_2_-Induced HepG2 Cells

#### 3.7.1. Construction of an Oxidative Damage Model

**Figure 4:**
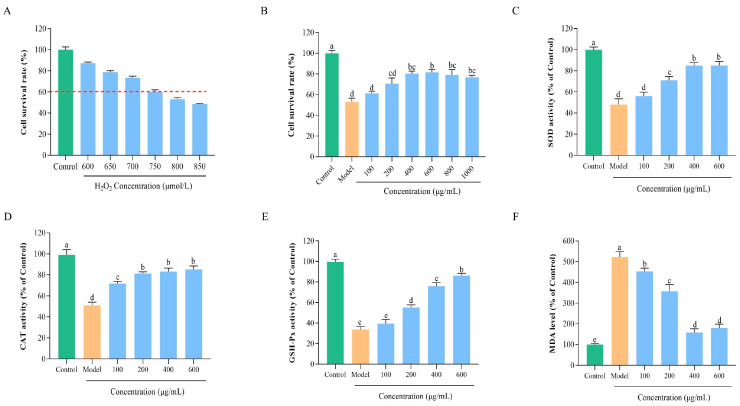
The protective effect and mechanism of BLP-1 on H_2_O_2_-induced HepG2 cells; (**A**) Screening of H_2_O_2_-induced concentration; (**B**) Effect of BLP-1 on the viability of HepG2 cells under oxidative stress; Effect of BLP-1 on the activities of antioxidant enzymes SOD (**C**), CAT (**D**), GSH-Px (**E**), and the level of MDA (**F**) in HepG2 cells under oxidative stress. All results were triplicates of the mean ± SD. Different lowercase letters represent significant difference (*p* < 0.05).

To further validate the antioxidant effects of BLP-1 at the cellular level, we constructed a cellular oxidative damage model to assess its protective effects on cells. In this study, HepG2 cells, which serve as a substitute for primary stem cells in biomedical research, were utilized to model oxidative damage [50]. Furthermore, H_2_O_2_ was employed as an inducer, as it stimulates ROS production and disrupts cellular redox balance [51]. As illustrated in Figure 4A, the cell viability of H_2_O_2_-induced HepG2 cells significantly decreased compared to the control group, exhibiting a dose-dependent effect. These results confirm the successful establishment of the oxidative damage model. Notably, when the concentration of H_2_O_2_ reached 750 μmol/L, the cell viability was measured at 53.17 ± 1.24%. It is widely accepted that a cell viability of approximately 50% represents the optimal condition for constructing a cellular oxidative damage model [52]. Therefore, a concentration of 750 μmol/L H_2_O_2_ was selected for subsequent experiments.

#### 3.7.2. Protective Effect of BLP-1 on Oxidatively Damaged HepG2 Cells

Under the condition of 750 μmol/L H_2_O_2_ induction, the protective effects of various concentrations of BLP-1 on HepG2 cells subjected to oxidative damage were investigated. As illustrated in Figure 4B, BLP-1 significantly alleviated oxidative stress-induced damage, with the protective effect increasing in correlation with BLP-1 concentration, peaking at 600 μg/mL, where cell viability was restored to 82.17 ± 2.23%. However, further increases in BLP-1 concentration did not enhance protective effects and even led to a decrease in cell viability, which aligns with findings reported by Kong and Hu et al. [48,53]. Given that BLP-1 is non-toxic and possesses growth-promoting properties, it is hypothesized that the observed reduction in cell viability at high peptide concentrations is not due to cytotoxicity. Instead, this reduction may result from the saturation of cell membrane receptors and transporters, as well as the disruption of intracellular homeostasis caused by excessively high peptide concentrations [54,55]. Consequently, BLP-1 concentrations of 100, 200, 400, and 600 μg/mL were selected for subsequent experiments.

#### 3.7.3. Regulation of BLP-1 on Antioxidant Enzyme Activity and MDA Levels

Within living organisms, a sophisticated and intricate antioxidant defense system exists, wherein endogenous antioxidant enzymes such as CAT, SOD, and GSH-Px play crucial roles in combating oxidative stress [56]. Specifically, SOD scavenges superoxide radicals to protect cells from oxidative damage, while CAT is essential for the decomposition of H_2_O_2_ [57]. Additionally, GSH-Px facilitates the reaction between H_2_O_2_ and GSH, leading to the generation of glutathione disulfide and the reduction in lipid peroxides to less harmful alcohols [58]. Therefore, measuring the activity of antioxidant enzymes in oxidative stress cells treated with BLP-1 is significant for understanding how BLP-1 exerts its protective effects against oxidative stress. As shown in Figure 4C–E, the cellular activities of SOD, CAT, and GSH-Px in the model group were significantly decreased compared to the control group. Following treatment with BLP-1, the cellular viabilities of these enzymes increased significantly in a dose-dependent manner, restoring to 85.05 ± 3.65%, 85.15 ± 3.40%, and 88.78 ± 1.38% of the control levels, respectively.

MDA is a primary end product of lipid peroxidation, formed when free radicals attack polyunsaturated fatty acids in cell membranes [59]. It serves as a marker for cell membrane damage and the extent of oxidative stress in the body [60]. As illustrated in Figure 4F, the MDA level in the model group was significantly elevated. However, after treatment with varying concentrations of BLP-1, the intracellular MDA content decreased markedly, exhibiting a negative correlation with BLP-1 concentration within a specific range (100–400 μg/mL). This suggests that BLP-1 effectively inhibits MDA production in cells subjected to oxidative stress. The above results indicate that BLP-1 can alleviate cellular oxidative damage caused by oxidative stress by enhancing the activity of antioxidant enzymes and reducing MDA levels, which aligns with previous research findings regarding the role of antioxidant peptides in mitigating cellular oxidative damage [61].

### 3.8. Protective Effect and Mechanism of BLP-1 on Mice with ALD

#### 3.8.1. Effect of BLP-1 on Liver Index in ALD Mice

**Figure 5:**
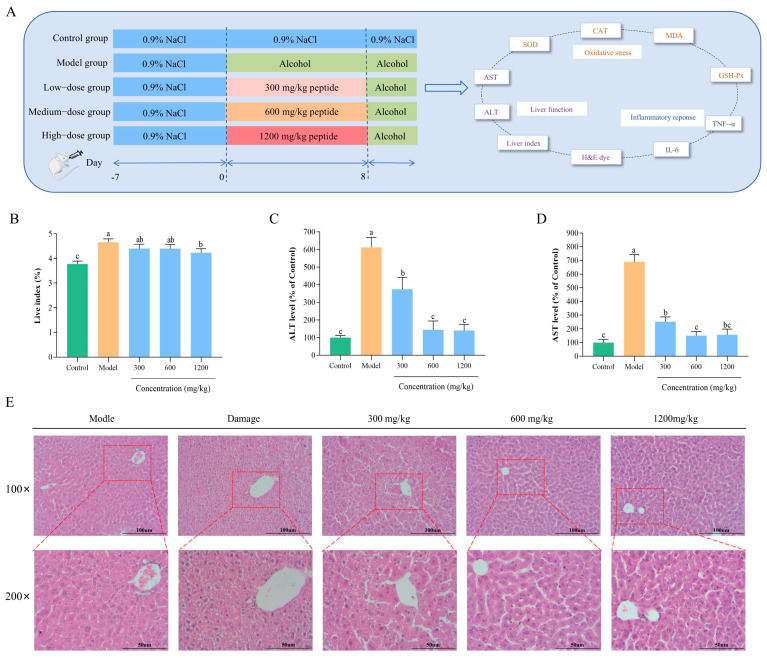
The hepatoprotective effect of BLP-1 on acute ALD in mice. (**A**) Schematic diagram of animal experiment design process; (**B**) Liver index; Effects of different concentrations of BLP-1 on ALT (**C**) and AST (**D**) transaminases in ALD mice; (**E**) H&E staining of liver sections from ALD mice in different treatment groups. All results were triplicates of the mean ± SD. Different lowercase letters represent significant difference (*p* < 0.05). (**A**) was partly generated using Servier Medical Art (licensed under CC-BY 3.0).

The liver index is a critical indicator for evaluating the extent of liver damage and serves as a fundamental basis for determining the successful establishment of the ALD model, as well as the protective effects of the drug [62]. The liver index results for each group are illustrated in Figure 5B. Compared to the control group, the liver index in the model group exhibited a significant increase, confirming the successful establishment of the model. Following treatment with varying concentrations of BLP-1, the liver indices in all treatment groups were notably lower than those in the model group, indicating that BLP-1 effectively mitigates the damage caused by ALD.

#### 3.8.2. Changes in Transaminase Levels in ALD Mice

Cellular damage leads to the release of ALT and AST into the bloodstream. Consequently, ALT and AST serve as critical molecular markers for assessing hepatocyte injury [63]. As illustrated in Figure 5C,D, the levels of ALT and AST in the model group increased by 6 to 7 times compared to the control group, indicating the development of severe ALD in the mice. Following intervention with BLP-1, the levels of ALT and AST significantly decreased in comparison to the model group. Notably, when the peptide concentration reached 600 mg/kg and 1200 mg/kg, no significant difference in ALT and AST levels was observed compared to the control group. These findings demonstrate that BLP-1 exerts a significant protective effect on the liver in mice with ALD.

#### 3.8.3. Histopathological Analysis After BLP-1 Treatment

To more intuitively observe the morphological changes in liver tissue due to alcoholic liver injury and subsequent peptide intervention, HE staining analysis was employed to further confirm the protective effect of BLP-1 on the liver (Figure 5E). In the control group, hepatocytes exhibited normal size, uniform shape, and a ruddy color with abundant cytoplasm. The cell boundaries and nuclei were distinct, and there was no evidence of edema or vacuolation, reflecting the histological characteristics of a healthy liver. Conversely, the model group demonstrated severe liver injury, characterized by significant swelling, vacuolar degeneration, nuclear displacement, and lipid droplet accumulation [64,65]. In the BLP-1 treatment group, an increase in peptide concentration was associated with a gradual decrease in the degree of pathological damage in the liver. This improvement was primarily evidenced by the normalization of cell size and morphology, a reduction in cell swelling and vacuolar degeneration, and a significant decrease in lipid droplet accumulation. These histomorphological observations provide direct confirmation of the protective effect of BLP-1 against ALD.

#### 3.8.4. BLP-1 Inhibits Hepatic Oxidative Stress

**Figure 6:**
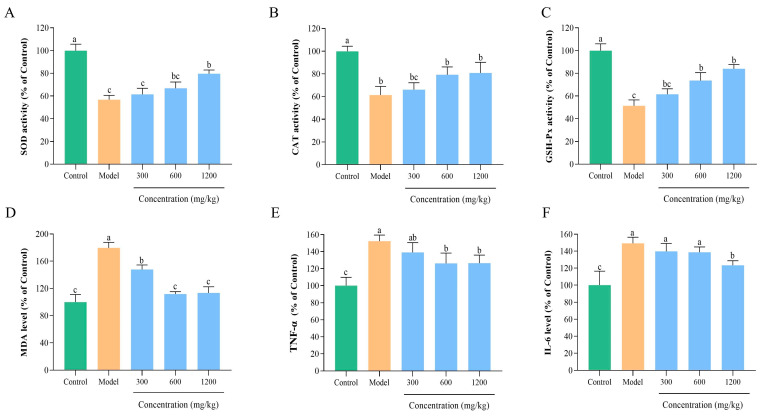
BLP-1 can alleviate oxidative stress and reduce the release of inflammatory factors. (**A**) SOD; (**B**) CAT; (**C**) GSH-Px; (**D**) MDA; (**E**) TNF-α; (**F**) IL-6. All results were triplicates of the mean ± SD. Different lowercase letters represent significant difference (*p* < 0.05).

Oxidative stress is a critical factor in the occurrence and progression of ALD, triggered by the excessive accumulation of ROS and the dysfunction of antioxidant defense mechanisms, both of which synergistically impair the integrity of liver structure and function [66]. To elucidate the protective mechanism of BLP-1 against ALD-associated hepatic oxidative stress, this study evaluated the activities of antioxidant enzymes, including SOD, CAT, and GSH-Px, as well as the level of MDA (Figure 6A–D). Compared to the control group, the ALD model group exhibited significantly reduced activities of SOD, CAT, and GSH-Px, along with a significantly elevated level of MDA, confirming the successful establishment of the model. Following treatment with peptides at concentrations of 300, 600, and 1200 mg/kg, oxidative stress was alleviated in a dose-dependent manner. Notably, the 1200 mg/kg peptide significantly restored the activities of SOD, CAT, and GSH-Px while reducing MDA levels. These findings indicate that BLP-1 alleviates hepatic oxidative stress in ALD mice by enhancing antioxidant enzyme activities and inhibiting lipid peroxidation, thereby exerting a hepatoprotective effect. This provides important mechanistic insights into the therapeutic potential of BLP-1 in ALD.

#### 3.8.5. BLP-1 Decreased Inflammatory Responses

Oxidative stress in ALD not only induces liver injury but also triggers an inflammatory cascade, with inflammation serving as another critical factor that exacerbates the progression of ALD [67,68]. This study aimed to investigate the anti-inflammatory effects of BLP-1 on the liver in ALD mice by conducting a quantitative analysis of inflammatory factors, including TNF-α and IL-6 (Figure 6E,F). Compared to the control group, the levels of TNF-α and IL-6 in the ALD model group were significantly elevated, indicating a severe imbalance in the regulation of liver inflammation. Treatment with peptides at concentrations of 300, 600, and 1200 mg/kg demonstrated a concentration-dependent anti-inflammatory effect, with the most pronounced effect observed in the 1200 mg/kg group. Consistent with the trend of BLP-1 reducing oxidative stress, these findings collectively suggest that BLP-1 can alleviate hepatic inflammatory responses in ALD mice. The underlying mechanism may be linked to the blockade of the oxidative stress-inflammation connection, providing critical mechanistic evidence for the therapeutic effects of BLP-1 in ALD intervention. Furthermore, the in vivo safety of BLP-1 in the treatment of ALD necessitates further investigation.

### 3.9. Screening of Antioxidant Peptides in BLP-1

#### 3.9.1. Screening Using PeptideRanker and BIOPEP Databases

**Figure 7:**
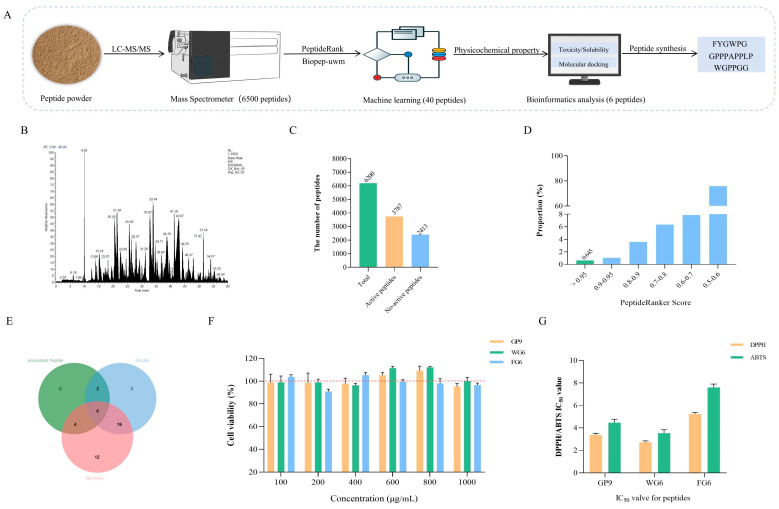
Screening of antioxidant peptide sequences in BLP-1 and detection of synthetic peptide indicators. (**A**) Flowchart of bioinformatics screening for antioxidant peptides. (**B**) The LC-MS/MS analysis of BLP-1; (**C**) The number of bioactive peptides in BLP-1; (**D**) Score proportion of PeptideRanker; (**E**) Venn diagram for screening antioxidant peptides with non-toxic and soluble characteristics. (**F**) Cytotoxicity of GP9, FG6, and WG6; (**G**) The DPPH/ABTS IC_50_ values of GP9, FG6, and WG6. All results were triplicates of the mean ± SD.

The screening process for antioxidant peptide sequences in BLP-1 is illustrated in Figure 7A. Currently, the PeptideRanker and BIOPEP databases are widely utilized for screening various bioactive peptides, including antioxidant peptides, angiotensin-converting enzyme (ACE) inhibitory peptides, and dipeptidyl peptidase-IV (DPP-IV) inhibitory peptides [69,70,71]. In this study, 6200 peptide sequences with a molecular weight ranging from 200 to 2500 Da were identified from BLP-1 through LC-MS/MS analysis (Figure 7B). Subsequently, the probability of these peptide sequences being bioactive was predicted using PeptideRanker [72]. Generally, a peptide sequence score exceeding 0.5 is considered indicative of a bioactive peptide, resulting in a total of 3787 sequences (Figure 7C) [73]. To enhance the accuracy of bioactive peptide screening, the PeptideRanker screening score threshold was set at 0.95. It was observed that the proportion of peptide sequences with scores greater than 0.95 was approximately 0.645%, corresponding to 40 peptide sequences (Figure 7D, Supplementary Table S3). Finally, 12 antioxidant peptides were identified from these 40 bioactive peptides using the BIOPEP database (Supplementary Table S4). It is noteworthy that during the screening process, 40 peptides with scores greater than 0.95 were identified. These peptides not only demonstrated antioxidant activity but also exhibited multiple biological activities, including ACE inhibition, dipeptidyl peptidase III inhibition, ACE2 inhibition, and pseudolysin inhibition. The additional biological activities of these peptides warrant further exploration in future research.

#### 3.9.2. Prediction of Peptide Physicochemical Properties

To further screen the peptide sequences, the physicochemical properties, including solubility and cytotoxicity of 12 antioxidant peptides were predicted, with the results presented in Table 2. The relative molecular weights of these peptides ranged from 500 to 1500 Da, which aligns with the findings of Li et al. [74] regarding the molecular weight distribution of antioxidant peptides. The GRAVY value serves as an indicator of peptide solubility, where a GRAVY value less than 0 suggests good water solubility, and a value greater than 0 indicates poor solubility [75]. Among the peptides, KF6, FW7, GL8, and MF6, which had GRAVY values exceeding 0, demonstrated poor solubility and were excluded from further screening. Additionally, FP7 and PG16 were predicted to possess certain levels of toxicity and were also excluded from subsequent evaluations. Consequently, out of the 40 bioactive peptide sequences with PeptideRanker scores greater than 0.95, 6 sequences were identified that exhibit antioxidant activity, are non-toxic, and possess good solubility (Figure 7E). These sequences are MP6, FG6, DW6, WG6, DGG6, and GP9.

#### 3.9.3. Molecular Docking with DPPH/ABTS

Further screening of the sequences was conducted by assessing the binding energy of peptides with DPPH and ABTS. The binding energy of six antioxidant peptides with DPPH ranged from −3.4 to −4.3 kcal/mol, and with ABTS, it also ranged from −3.4 to −4.3 kcal/mol (Table 3, Supplementary Figure S1). This range of binding energy aligns with the findings reported by Liu et al. regarding the docking binding energy of corn cyclic peptides with DPPH and ABTS [29]. A docking binding energy of less than 0 indicates that the ligand and receptor can spontaneously bind, suggesting that all antioxidant peptides are capable of spontaneous binding with both DPPH and ABTS [76]. Moreover, a lower docking score correlates with greater binding affinity. By integrating the binding capabilities of the antioxidant peptides with DPPH and ABTS, their antioxidant capacities were ranked as follows: GP9 > FG6 > WG6 > DGG6 > DW6 > MP6. Consequently, GP9, FG6, and WG6 were selected for synthesis for further validation.

### 3.10. Peptide Synthesis and Validation

The MS of GP9, FG6, and WG6 are presented in Supplementary Figure S2. Cytotoxicity tests were conducted on the three antioxidant peptides, revealing that cell viability consistently exceeded 90% across concentrations ranging from 100 to 1000 μg/mL. This finding indicates that the three antioxidant peptides did not exhibit significant cytotoxicity (Figure 7F). Further assessment of the antioxidant activity of the three peptides showed that the IC50 values for DPPH scavenging were 3.37 ± 0.15, 2.73 ± 0.09, and 5.23 ± 0.15 mg/mL for GP9, FG6, and WG6, respectively. In addition, the IC50 values for ABTS scavenging were recorded as 4.47 ± 0.31, 3.53 ± 0.31, and 7.60 ± 0.30 mg/mL, respectively (Figure 7G). These values are comparable to the antioxidant capacity of the sequences derived from goat milk as reported by Zhang et al. [77].

### 3.11. Molecular Docking with KeaP1

**Figure 8:**
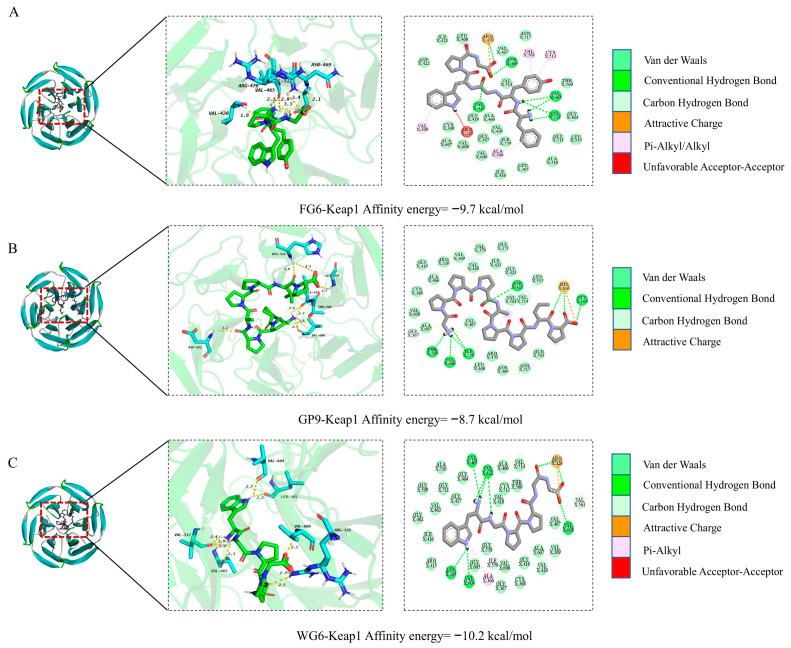
Molecular docking of antioxidant peptides with Keap1. (**A**) FG6; (**B**) GP9; (**C**) WG6.

In organisms, maintaining the dynamic balance of redox is a complex process that necessitates the coordinated functioning of multiple signaling pathways. Among these, the Nrf2/Keap1 signaling pathway plays a crucial role in maintaining cellular homeostasis and responding to oxidative stress [76]. Consequently, molecular docking studies were conducted between FG6, GP9, WG6, and Keap1 to investigate the protective mechanisms of these three antioxidant peptides (Figure 8). Docking binding energy is a widely used metric for evaluating the binding affinity between a receptor and a ligand, with lower binding energy indicating stronger affinity [78]. The binding energies of the three antioxidant peptides with Keap1 were −9.7, −8.7, and −10.2 kcal/mol, respectively. Yang et al. suggest that a docking binding energy of less than −7 kcal/mol indicates a strong binding affinity between the ligand and the receptor. Therefore, these three antioxidant peptides demonstrate a robust binding capability to Keap1. Based on the experimental results, FG6, GP9, and WG6 may influence the interaction between Keap1 and Nrf2 by binding to Keap1, which leads to the dissociation and release of Nrf2, thereby affecting the expression of downstream genes and alleviating oxidative stress-induced damage.

## 4. Conclusions

This study utilized bovine lung as the raw material and established an extraction process centered on papain through single-factor optimization combined with response surface methodology, successfully obtaining the highly antioxidative active component BLP-1. In vitro experiments demonstrated that BLP-1 exhibits strong scavenging capabilities against DPPH, ABTS, hydroxyl, and superoxide anion radicals, with no significant cytotoxicity or hemolytic activity. In the HepG2 cell oxidative stress model, BLP-1 significantly enhanced cell viability by restoring the activities of SOD, CAT, and GSH-Px while inhibiting MDA generation. Animal experiments further confirmed that BLP-1 could reduce serum ALT and AST levels in ALD mice, inhibit the release of TNF-α and IL-6, and ameliorate pathological liver tissue damage. Furthermore, through LC-MS/MS combined with bioinformatics analysis, 12 novel antioxidant peptide sequences were identified, among which GP9, FG6, and WG6 exhibited strong binding affinity to Keap1, suggesting their potential to activate the antioxidant defense system by competitively inhibiting the Keap1-Nrf2 interaction. This study systematically explored the extraction process, bioactivity, and protective effects, as well as the mechanisms of the bovine lung-derived antioxidant peptide BLP-1 against ALD. However, although this study confirmed the antioxidant effects of BLP-1 on oxidative damage in cells and its protective role against ALD, it has not yet been verified whether the identified peptides exhibit the same efficacy. Future research should focus on peptide synthesis in conjunction with cellular or animal experiments for comprehensive validation. Additionally, this study only verified the antioxidant pathway of the identified peptides through molecular docking, and further experimental validation is still required.

## Figures and Tables

**Table 1:** Analysis of variance of the regression model for DPPH/ABTS clearance rate.

Source	DPPH Clearance Rate (%)						ABTS Clearance Rate (%)					
	Sum of Squares	df	Mean Square	F-Value	*p*-Value	Significance	Sum of Squares	df	Mean Square	F-Value	*p*-Value	Significance
Model	100.71	9	11.19	39.87	<0.0001	Yes	104.28	9	11.59	28.45	0.0001	Yes
A	4.31	1	4.31	15.35	0.0058		1.19	1	1.19	2.91	0.1317	
B	5.99	1	5.99	21.33	0.0024		4.99	1	4.99	12.26	0.0100	
C	2.13	1	2.13	7.60	0.0283		3.54	1	3.54	8.69	0.0215	
AB	3.48	1	3.48	12.39	0.0097		0.6806	1	0.6806	1.67	0.2371	
AC	2.04	1	2.04	7.29	0.0307		2.42	1	2.42	5.94	0.0450	
BC	0.06	1	0.06	0.2139	0.6578		0.1332	1	0.1332	0.3271	0.5852	
A^2^	38.84	1	38.84	138.37	<0.0001		37.00	1	37.00	90.85	<0.0001	
B^2^	24.65	1	24.65	87.85	<0.0001		15.43	1	15.43	37.89	0.0005	
C^2^	11.15	1	11.15	39.71	0.0004		29.66	1	29.66	75.84	<0.0001	
Residual	1.96	7	0.2807				2.85	7	0.4072			
Lack of Fit	1.50	3	0.4998	4.30	0.0965	No	1.60	3	0.5342	1.71	0.3017	No
Pure Error	0.4653	4	0.1163				1.25	4	0.3120			
Cor Total	102.68	16					107.13	16				
	R^2^ = 0.9809			R_ad_j^2^ = 0.9563			R^2^ = 0.9734			R_adj_^2^ = 0.9392		
CV%	0.6311						0.8276					

**Table 2:** Prediction of Physicochemical Properties of Antioxidant Peptides.

Name	Sequence	Pepitidebank Score	Sequence	Charge	Mw	Toxin	GRAVY
KF6	KPFPFF	0.992914	6	1	781.95	Non-Toxin	0.217
MP6	MWPPLP	0.980232	6	0	739.94	Non-Toxin	−2.96
FG6	FYGWPG	0.978696	6	0	725.80	Non-Toxin	−0.300
DW6	DGGGWW	0.970555	6	−1	676.69	Non-Toxin	−1.083
FP7	FGPPPPP	0.969438	7	0	707.83	Toxin	−0.800
WG6	WGPPGG	0.959932	6	0	569.62	Non-Toxin	−0.883
FW7	FFSPGVW	0.95771	7	0	838.96	Non-Toxin	0.871
PG16	PPPGPPPPPGPPPPPG	0.955487	16	0	1451.69	Toxin	−1.375
DGG6	DGGAWW	0.954871	6	−1	690.71	Non-Toxin	−0.717
GL8	GWNIPMGL	0.953627	8	0	887.07	Non-Toxin	0.425
GP9	GPPPAPPLP	0.953422	9	0	842.00	Non-Toxin	−0.489
MF6	MIKPFF	0.95295	6	1	782.02	Non-Toxin	1.083

**Table 3:** Statistics of the molecular docking results between antioxidant peptides and DPPH/ABTS.

Name	DPPH			ABTS		
	Binding Energy (kcal/mol)	Conventional Hydrogen Bond	Binding Site	Binding Energy (kcal/mol)	Conventional Hydrogen Bond	Binding Site
MP6	−3.4	0	/	−3.4	1	Met1
FG6	−4.0	0	/	−4.3	2	Trp4
DW6	−3.6	2	Trp5, Trp6	−3.6	/	/
WG6	−3.9	2	Gly2, Gly6	−3.6	2	Pro3, Gly5
DGG6	−3.4	0	/	−3.9	3	Ala4, Trp5, Trp6
GP9	−4.3	2	Ala5, Leu8	−4.3	2	Leu8

## Data Availability

The data of this study are available from the corresponding authors upon reasonable request.

## Supplementary Materials

The following supporting information can be downloaded at https://www.mdpi.com/article/10.3390/antiox14111314/s1, Figure S1: Two-dimensional docking diagram of antioxidant peptides with DPPH/ABTS; Figure S2: Mass spectrum of antioxidant peptides. A: FYGWPG; B: GPPPAPPLP; C: WGPPGG; Table S1: Factors and levels in response surface design; Table S2: RSM experimental design and results for DPPH/ABTS clearance rate; Table S3: Peptide sequences with a pepitidebank score above 0.95; Table S4: Peptide bioactivity prediction.

## References

[1] Rehm J., Guiraud J., Poulnais R., Shield K.D. (2018). Alcohol dependence and very high risk level of alcohol consumption: A life-threatening and debilitating disease. Addict. Biol..

[2] Giuliano C., Goodlett C.R., Economidou D., García-Pardo M.P., Belin D., Robbins T.W., Bullmore E.T., Everitt B.J. (2015). The Novel μ-Opioid Receptor Antagonist GSK1521498 Decreases Both Alcohol Seeking and Drinking: Evidence from a New Preclinical Model of Alcohol Seeking. Neuropsychopharmacol. Off. Publ. Am. Coll. Neuropsychopharmacol..

[3] Mason B.J., Quello S., Goodell V., Shadan F., Begovic A. (2014). Gabapentin treatment for alcohol dependence: A randomized clinical trial. JAMA Intern. Med..

[4] Evangelou E., Suzuki H., Bai W., Pazoki R., Elliott P. (2021). Alcohol consumption in the general population is associated with structural changes in multiple organ systems: A population-based study in UK Biobank. eLife Sci..

[5] Addolorato G., Mirijello A., Barrio P., Gual A. (2016). Treatment of alcohol use disorders in patients with alcoholic liver disease. J. Hepatol..

[6] Ina B., Craig J.M., Gavin E.A. (2005). Treatment of Alcoholic Liver Disease. Dig. Dis..

[7] Zakhari S. (2006). Overview: How is alcohol metabolized by the body?. Alcohol Res. Health J. Natl. Inst. Alcohol Abus. Alcohol..

[8] Kim D.K., Kim Y.H., Jang H.H., Park J., Kim J.R., Koh M., Jeong W.I., Koo S.H., Park T.S., Yun C.H. (2013). Estrogen-related receptor γ controls hepatic CB1 receptor-mediated CYP2E1 expression and oxidative liver injury by alcohol. Gut.

[9] Peng H.C., Chen Y.L., Yang S.Y. (2013). The antiapoptotic effects of different doses of β-carotene in chronic ethanol-fed rats. HepatoBiliary Surg. Nutr..

[10] Lai W., Zhang J., Sun J., Min T., Bai Y., He J., Cao H., Che Q., Guo J., Su Z. (2024). Oxidative stress in alcoholic liver disease, focusing on proteins, nucleic acids, and lipids: A review. Int. J. Biol. Macromol..

[11] Xu J., Ma H.Y., Liang S., Sun M., Karin G., Koyama Y., Hu R., Quehenberger O., Davidson N.O., Dennis E.A. (2017). The role of human cytochrome P450 2E1 in liver inflammation and fibrosis. Hepatol. Commun..

[12] Liang J., Liu Y., Liu J., Li Z., Fan Q., Jiang Z., Yan F., Wang Z., Huang P., Feng N. (2018). Chitosan-functionalized lipid-polymer hybrid nanoparticles for oral delivery of silymarin and enhanced lipid-lowering effect in NAFLD. J. Nanobiotechnol..

[13] Soleimani V., Delghandi P.S., Moallem S.A., Karimi G. (2019). Safety and toxicity of silymarin, the major constituent of milk thistle extract: An updated review. Phytother. Res. PTR.

[14] Grădinariu L., Dediu L., Crețu M., Grecu I.R., Docan A., Istrati D.I., Dima F.M., Stroe M.D., Vizireanu C. (2024). The Antioxidant and Hepatoprotective Potential of Berberine and Silymarin on Acetaminophen Induced Toxicity in *Cyprinus carpio* L.. Animals.

[15] Xu W., Lu H., Yuan Y., Deng Z., Zheng L., Li H. (2022). The Antioxidant and Anti-Inflammatory Effects of Flavonoids from Propolis via Nrf2 and NF-κB Pathways. Foods.

[16] Tonolo F., Folda A., Scalcon V., Marin O., Bindoli A., Rigobello M.P. (2022). Nrf2-Activating Bioactive Peptides Exert Anti-Inflammatory Activity through Inhibition of the NF-κB Pathway. Int. J. Mol. Sci..

[17] Ana P.C.C., Francisco E.S.L., Francisca F.N.A., Yago O.P., Claudia R.A., Felipe P.M., Gabrielly O.S., Cleverson D.T.F., Pedro F.N.S. (2023). Antioxidant peptides from plants: A review. Phytochem. Rev..

[18] Mao Z., Jiang H., Sun J., Zhao Y., Gao X., Mao X. (2024). Research progress in the preparation and structure-activity relationship of bioactive peptides derived from aquatic foods. Trends Food Sci. Technol..

[19] Lu W.C., Chiu C.S., Chan Y.J., Guo T.P., Lin C.C., Wang P.C., Lin P.Y., Mulio A.T., Li P.H. (2022). An In Vivo Study to Evaluate the Efficacy of Blue Shark (*Prionace glauca*) Cartilage Collagen as a Cosmetic. Mar. Drugs.

[20] Leticia M., Milagro R., Fidel T. (2014). Bioactive peptides generated from meat industry by-products. Food Res. Int..

[21] Jayawardena S.R., Morton J.D., Brennan C.S., Bekhit A.E.-D.A. (2019). Utilisation of beef lung protein powder as a functional ingredient to enhance protein and iron content of fresh pasta. Int. J. Food Sci. Technol..

[22] Jayawardena S.R., Morton J.D., Bekhit A.E.-D.A., Bhat Z.F., Brennan C.S. (2022). Effect of drying temperature on nutritional, functional and pasting properties and storage stability of beef lung powder, a prospective protein ingredient for food supplements. LWT.

[23] O’SUllivan S.M., Lafarga T., Hayes M., O’BRien N.M. (2017). Bioactivity of bovine lung hydrolysates prepared using papain, pepsin, and Alcalase. J. Food Biochem..

[24] Wei G., Zhao Q., Wang D., Fan Y., Shi Y., Huang A. (2022). Novel ACE inhibitory, antioxidant and α-glucosidase inhibitory peptides identified from fermented rubing cheese through peptidomic and molecular docking. LWT.

[25] Fan X., Han Y., Sun Y., Zhang T., Tu M., Du L., Pan D. (2023). Preparation and characterization of duck liver-derived antioxidant peptides based on LC-MS/MS, molecular docking, and machine learning. LWT.

[26] Xiao X., Meng J., Chen X., Li Y., Zheng S., Zhang X., Zhang L., Fang J., Su T., Ma Z. (2025). Preparation, Identification, and Cellular Antioxidant Activity Evaluation of Antioxidant Peptides from the Antler Tips of Sika Deer (*Cervus nippon*). J. Food Biochem..

[27] Chen X., Xia P., Zheng S., Li Y., Fang J., Ma Z., Zhang L., Zhang X., Hao L., Zhang H. (2023). Antioxidant Peptides from the Collagen of Antler Ossified Tissue and Their Protective Effects against H_2_O_2_-Induced Oxidative Damage toward HaCaT Cells. Molecules.

[28] Su T., Liu L., Lv Q., Zhang X., Hao L., Lv Y., Song Q. (2024). Preparation of Bovine Bone Collagen Peptide Wine and Its Protective Effect on Alcoholic Liver Injury in Mice. J. Jilin Agric. Univ..

[29] Liu H., Fan H., Teng X., Sun T., Zhang S., Wang N., Zhang X., Liu T., Zhang Y., Wang D. (2025). Exploring novel antioxidant cyclic peptides in corn protein hydrolysate: Preparation, identification and molecular docking analysis. Food Chem..

[30] Hu X., Liu J., Li J., Song Y., Chen S., Zhou S., Yang X. (2022). Preparation, purification, and identification of novel antioxidant peptides derived from *Gracilariopsis lemaneiformis* protein hydrolysates. Front. Nutr..

[31] Neha S., Gily N., Tenzin T., Daniel N.A.B. (2022). Sequence dependencies and biophysical features both govern cleavage of diverse cut-sites by HIV protease. Protein Sci..

[32] Sun K.-L., Gao M., Wang Y.-Z., Li X.-R., Wang P., Wang B. (2022). Antioxidant Peptides From Protein Hydrolysate of Marine Red Algae *Eucheuma cottonii*: Preparation, Identification, and Cytoprotective Mechanisms on H_2_O_2_ Oxidative Damaged HUVECs. Front. Microbiol..

[33] Kim E.-K., Lee S.-J., Jeon B.-T., Moon S.-H., Kim B., Park T.-K., Han J.-S., Park P.-J. (2009). Purification and characterisation of antioxidative peptides from enzymatic hydrolysates of venison protein. Food Chem..

[34] Lee W.-S., Jeon J.-K., Byun H.-G. (2011). Characterization of a novel antioxidative peptide from the sand eel *Hypoptychus dybowskii*. Process Biochem..

[35] Ji Y., Zhang G., Li X., Zhao B., Zhou S. (2013). Enzymatic hydrolysis of protein from small yellow croaker (*Psendosciaena polyactis*) and evaluation of its antioxidant activity. J. Food Biochem..

[36] Esfandi R., Willmore W.G., Tsopmo A. (2019). Peptidomic analysis of hydrolyzed oat bran proteins, and their in vitro antioxidant and metal chelating properties. Food Chem..

[37] Tan Y., Wang Y., Wan Y., Liang Y., Liu Q., Wei M., Hou T. (2024). Preparation, Structural Identification, and Screening of Egg-Derived Peptides with Facilitating Alcohol Metabolism Activity. Foods.

[38] Jin Y., Zhou P., Zhu C., Liu Y., Zhao Z. (2024). Preparation of Antioxidant Peptides from Chicken Bone Proteins and the Influence of Their Compositional Characteristics on Antioxidant Activity. Foods.

[39] Xie Z., Wang X., Yu S., He M., Yu S., Xiao H., Song Y. (2021). Antioxidant and functional properties of cowhide collagen peptides. J. Food Sci..

[40] Chen B., Xia Z., Ye H., Wang Q., Huang W., Zhang L., Chen W., Liang D., Liang X., Yin Y. (2023). Response surface optimization of selenium-enriched *Moringa oleifera* seed peptides with antioxidant, ACEI and XOI activities. J. Food Meas. Charact..

[41] Liu Q., Xun G., Feng Y. (2019). The state-of-the-art strategies of protein engineering for enzyme stabilization. Biotechnol. Adv..

[42] Gao D., Chen H., Liu H., Yang X., Guo P., Cao X., Cai Y., Xu H., Yang J. (2022). Structure characterization and antioxidant activity analysis of polysaccharides from Lanzhou Lily. Front. Nutr..

[43] Yuan E., Nie S., Liu L., Ren J. (2021). Study on the interaction of *Hericium erinaceus* mycelium polysaccharides and its degradation products with food additive silica nanoparticles. Food Chem. X.

[44] Maria I., Elisavet B., Stamatia C., Adriana S., Paschalina C. (2023). Modeling and Optimization of Phenolic Compounds from Sage (*Salvia fruticosa* L.) Post-Distillation Residues: Ultrasound- versus Microwave-Assisted Extraction. Antioxidants.

[45] Wang L., Li Z., Fan X., Zhang T., Wang H., Ye K. (2024). Novel antioxidant peptides from bovine blood: Purification, identification and mechanism of action. LWT.

[46] Yang W. (2024). Evaluation of the antioxidant activity and identification of potential antioxidant peptides in commercially available probiotic Cheddar cheese. LWT.

[47] Jin H.-X., Xu H.-P., Li Y., Zhang Q.-W., Xie H. (2019). Preparation and Evaluation of Peptides with Potential Antioxidant Activity by Microwave Assisted Enzymatic Hydrolysis of Collagen from Sea Cucumber Acaudina Molpadioides Obtained from Zhejiang Province in China. Mar. Drugs.

[48] Hu Y.-M., Lu S.-Z., Li Y.-S., Wang H., Shi Y., Zhang L., Tu Z.-C. (2022). Protective effect of antioxidant peptides from grass carp scale gelatin on the H2O2-mediated oxidative injured HepG2 cells. Food Chem..

[49] Li Y., Jiao H., Zhang H., Wang X., Fu Y., Wang Q., Liu H., Yong Y., Guo J., Liu J. (2024). Biosafety consideration of nanocellulose in biomedical applications: A review. Int. J. Biol. Macromol..

[50] Asadian S., Piryaei A., Gheibi N., Kalantari B.A., Davarpanah M.R., Azad M., Kapustina V., Alikhani M., Nejad S.M., Alikhani H.K. (2022). Rhenium Perrhenate ((188)ReO(4)) Induced Apoptosis and Reduced Cancerous Phenotype in Liver Cancer Cells. Cells.

[51] He Y., Peng L., Xiong H., Liu W., Zhang H., Peng X., Zhu X., Guo F., Sun Y. (2023). The profiles of durian (*Durio zibethinus* Murr.) shell phenolics and their antioxidant effects on H_2_O_2_-treated HepG2 cells as well as the metabolites and organ distribution in rats. Food Res. Int..

[52] Xu Q., Liu M., Chao X., Zhang C., Yang H., Chen J., Zhao C., Zhou B. (2022). Acidifiers Attenuate Diquat-Induced Oxidative Stress and Inflammatory Responses by Regulating NF-κB/MAPK/COX-2 Pathways in IPEC-J2 Cells. Antioxidants.

[53] Kong Y., Feng M., Sun J. (2023). Novel antioxidant peptides in fermented pork sausage: Purification, characterization, and cytoprotective functions on Caco-2 cells. Food Chem..

[54] Taghikhani E., Fromm M.F., König J. (2017). Assays for Analyzing the Role of Transport Proteins in the Uptake and the Vectorial Transport of Substances Affecting Cell Viability. Methods Mol. Biol..

[55] Zhang L., Yang Z., Zeng W., Liang B., Huang Z., Wang Q., Li Z., Chen T., Yan B. (2025). Hierarchical Vanadium Detoxification Mechanisms in Gram-Positive *Enterococcus faecalis*: Extracellular Chelation, Antioxidant Defense, and Metabolic Reprogramming. ACS EST Eng..

[56] Li Y., Liu H., Zhang L., Yang Y., Lin Y., Zhuo Y., Fang Z., Che L., Feng B., Xu S. (2019). Maternal Dietary Fiber Composition during Gestation Induces Changes in Offspring Antioxidative Capacity, Inflammatory Response, and Gut Microbiota in a Sow Model. Int. J. Mol. Sci..

[57] Antoniou C., Xenofontos R., Chatzimichail G., Christou A., Kashfi K., Fotopoulos V. (2020). Exploring the Potential of Nitric Oxide and Hydrogen Sulfide (NOSH)-Releasing Synthetic Compounds as Novel Priming Agents against Drought Stress in Medicago sativa Plants. Biomolecules.

[58] Lim S.H., Choi C.I. (2021). Potentials of Raspberry Ketone as a Natural Antioxidant. Antioxidants.

[59] Shi L., Zheng Y., Cheng Z., Ji N., Niu C., Wang Y., Huang T., Li R., Huang M., Chen X. (2022). One-year follow-up study after patients with severe COVID-19 received human umbilical cord mesenchymal stem cells treatment. Stem Cell Res. Ther..

[60] Chen J., Shu Y., Chen Y., Ge Z., Zhang C., Cao J., Li X., Wang Y., Sun C. (2022). Evaluation of Antioxidant Capacity and Gut Microbiota Modulatory Effects of Different Kinds of Berries. Antioxidants.

[61] Xie R.-H., Xiao S., Ma D., Wang B., Chen G.-C., Xiang J.-H., Wang J.-H. (2024). Protective mechanism of antioxidant peptides derived from dry-cured ham against ultraviolet A-induced oxidative damage in HaCat cells. Food Biosci..

[62] Guo Q., Zhang L., Yin Y., Gong S., Yang Y., Chen S., Han M., Duan Y. (2022). Taurine Attenuates Oxidized Fish Oil-Induced Oxidative Stress and Lipid Metabolism Disorder in Mice. Antioxidants.

[63] de Stoppelaar S.F., van ‘t Veer C., Claushuis T.A., Albersen B.J., Roelofs J.J., van der Poll T. (2014). Thrombocytopenia impairs host defense in gram-negative pneumonia-derived sepsis in mice. Blood.

[64] Zhou X., Deng Q., Chen H., Hu E., Zhao C., Gong X. (2017). Characterizations and hepatoprotective effect of polysaccharides from Mori Fructus in rats with alcoholic-induced liver injury. Int. J. Biol. Macromol..

[65] Govindan S., Jayabal A., Shanmugam J., Ramani P. (2021). Antioxidant and hepatoprotective effects of *Hypsizygus ulmarius* polysaccharide on alcoholic liver injury in rats. Food Sci. Hum. Wellness.

[66] Mitchell R.M., Hartmut J. (2013). Oxidant Stress, Antioxidant Defense, and Liver Injury. Drug-Induced Liver Disease.

[67] Tilg H., Moschen A.R., Szabo G. (2016). Interleukin-1 and inflammasomes in alcoholic liver disease/acute alcoholic hepatitis and nonalcoholic fatty liver disease/nonalcoholic steatohepatitis. Hepatology.

[68] Tilg H., Moschen A.R., Kaneider N.C. (2011). Pathways of liver injury in alcoholic liver disease. J. Hepatol..

[69] Cerrato A., Aita S.E., Capriotti A.L., Cavaliere C., Montone A.M.I., Montone C.M., Laganà A. (2022). Investigating the Short Peptidome Profile of Italian Dry-Cured Ham at Different Processing Times by High-Resolution Mass Spectrometry and Chemometrics. Int. J. Mol. Sci..

[70] Chiozzi R.Z., Capriotti A.L., Cavaliere C., La Barbera G., Piovesana S., Samperi R., Laganà A. (2016). Purification and identification of endogenous antioxidant and ACE-inhibitory peptides from donkey milk by multidimensional liquid chromatography and nanoHPLC-high resolution mass spectrometry. Anal. Bioanal. Chem..

[71] Han R., Maycock J., Murray B.S., Boesch C. (2019). Identification of angiotensin converting enzyme and dipeptidyl peptidase-IV inhibitory peptides derived from oilseed proteins using two integrated bioinformatic approaches. Food Res. Int..

[72] Fang J., Lu J., Zhang Y., Wang J., Wang S., Fan H., Zhang J., Dai W., Gao J., Yu H. (2021). Structural properties, antioxidant and immune activities of low molecular weight peptides from soybean dregs (Okara). Food Chem. X.

[73] Iwaniak A., Minkiewicz P., Pliszka M., Mogut D., Darewicz M. (2020). Characteristics of Biopeptides Released In Silico from Collagens Using Quantitative Parameters. Foods.

[74] Li X.-X., Han L.-J., Chen L.-J. (2008). In vitro antioxidant activity of protein hydrolysates prepared from corn gluten meal. J. Sci. Food Agric..

[75] Chen J., Liu Y., Wang G., Sun S., Liu R., Hong B., Gao R., Bai K. (2018). Processing Optimization and Characterization of Angiotensin-Ι-Converting Enzyme Inhibitory Peptides from Lizardfish (*Synodus macrops*) Scale Gelatin. Mar. Drugs.

[76] Zhu Y., Zhu Y., Tao S., Liang W., Zhang J., Zhang Y., Xuan Z., Xu J., Peng C., Wu H. (2022). The Integrated Study on the Chemical Profiling to Explore the Constituents and Mechanism of Traditional Chinese Medicine Preparation Huatuo Jiuxin Pills Based on UPLC-Q-TOF/MS(E) and Network Pharmacology. Front. Mol. Biosci..

[77] Zhang W., Abubaker M.A., Li Z., He Y., Shu Q., Li L., Liu Y. (2025). Bioactive peptides with antioxidant and ACE inhibitory properties in goat milk protein hydrolysates: Peptidomics and molecular docking study. Int. J. Biol. Macromol..

[78] Chen M., Wen H., Zhou S., Yan X., Li H. (2022). Patchouli Alcohol Inhibits D-Gal Induced Oxidative Stress and Ameliorates the Quality of Aging Cartilage via Activating the Nrf2/HO-1 Pathway in Mice. Oxidative Med. Cell. Longev..

