# Supplementary material for: Extraction, Purification and Identification of Bovine Lung Peptides and Its Antioxidant Effects on H2O2-Induced HepG2 Cells and Mice with Alcoholic Liver Injury
Source: Antioxidants (Basel). 2025 Oct 31;14(11):1314. doi: 10.3390/antiox14111314 (PMC12649525; doi:10.3390/antiox14111314)
Supplement: Supplementary file 1 [file antioxidants-14-01314-s001.zip › antioxidants-3910315-supplementary.pdf]

Supplementary Table S1 Factors and levels in response surface design

Independent Factors	Symbol	Level of Factor		
Enzyme concentration (U/g)	A	3000	4000	5000
pH	B	7	8	9
Hydrolysis time (h)	C	3	4	5

Supplementary Table S2 RSM experimental design and results for DPPH/ABTS clearance rate

Run	A. Factor 1	B. Factor 2	C. Factor 3	Clearance rate (%)	
				DPPH	ABTS
1	5000	9	4	80.83	74.75
2	4000	8	4	87.06	80.70
3	4000	8	4	87.53	79.98
4	4000	8	4	86.83	80.23
5	3000	8	3	84.82	76.80
6	4000	9	3	82.84	75.59
7	4000	7	3	83.92	77.16
8	4000	8	4	87.67	81.34
9	5000	7	4	81.10	75.35
10	3000	9	4	80.67	75.38
11	3000	7	4	84.67	77.63
12	4000	7	5	83.86	76.65
13	4000	8	4	87.28	81.03
14	3000	8	5	81.63	73.36
15	5000	8	5	81.83	74.83
16	4000	9	5	82.29	74.65
17	5000	8	3	82.16	75.16

Supplementary Table S3 Peptide sequences with a pepitidebank score above 0.95

Sequence	Pepitidebank score
KPFPFF	0.992914
PPPFPPPG	0.984932
GPFPPPP	0.980356
MWPPLP	0.980232
FYGWPG	0.978696
PGPLPPF	0.976014
PGFPPPP	0.975811
MPGPLF	0.974116
FPAPFPASGARLC	0.972457
SHGGFF	0.971252
GDPNWF	0.97125
DGGGWW	0.970555
FGPPPPP	0.969438
PGIPPPF	0.969026
FDPFLR	0.96809
GDPNWFMK	0.965355
GPAPPPPLP	0.962485
IDGFFP	0.960357
WPGPGG	0.960077
WGPPGG	0.959932
WCQHYW	0.958757
FGGGGF	0.958643
FPKMIF	0.958126
PGMNPF	0.957929
FFSPGVW	0.95771
PGGGPF	0.956992
FPDFLR	0.956391
PPPGPPPPPGPPPPPG	0.955487
WTQRFF	0.955452
FSWSAF	0.955189
DPNWFPK	0.955176
DGGAWW	0.954871
PGGPGF	0.954306
GWNIPMGL	0.953627
GPPPAPPLP	0.953422
MIKPFF	0.95295
AGGPFP	0.951961
WTGSFF	0.951499
SFPGSF	0.951162
GPAGMPGFP	0.950793

Supplementary Table S4 Peptide bioactivity prediction

Sequence	Bioactivity.
KPFPFF	ACE inhibitor、antioxidative、dipeptidyl peptidase IV inhibitor、dipeptidyl peptidase III inhibitor、ACE2 inhibitor、pseudolysin inhibitor
PPPFPPPG	Antiamnestic、ACE inhibitor、antithrombotic、regulating、inhibitor、dipeptidyl peptidase IV inhibitor、alpha-glucosidase inhibitor、dipeptidyl peptidase III inhibitor、ACE2 inhibitor、PAM inhibitor
GPFPPPP	Antiamnestic、ACE inhibitor、antithrombotic、regulating、antioxidative、hypotensive、dipeptidyl peptidase IV inhibitor、alpha-glucosidase inhibitor、dipeptidyl peptidase III inhibitor、ACE2 inhibitor、neprilysin inhibitor
MWPPLP	ACE inhibitor、antioxidative、inhibitor、dipeptidyl peptidase IV inhibitor、alpha-glucosidase inhibitor、xaa-pro inhibitor、lactocepin inhibitor
FYGWPG	alpha-amylase inhibitor、antiamnestic、ACE inhibitor、antithrombotic、immunostimulating、regulating、antioxidative、hypotensive、dipeptidyl peptidase IV inhibitor、alpha-glucosidase inhibitor、dipeptidyl peptidase III inhibitor、PAM inhibitor、inhibitor of tripeptidyl peptidase II、tubulin-tyrosine ligase inhibitor
PGPLPPF	Antiamnestic、ACE inhibitor、antithrombotic、regulating、antioxidative、inhibitor、chemotactic、hypotensive、dipeptidyl peptidase IV inhibitor、alpha-glucosidase inhibitor、dipeptidyl peptidase III inhibitor、ACE2 inhibitor、PAM inhibitor、xaa-pro inhibitor、lactocepin inhibitor、neprilysin inhibitor
PGFPPPP	Antiamnestic、ACE inhibitor、antithrombotic、regulating、dipeptidyl peptidase IV inhibitor、alpha-glucosidase inhibitor、dipeptidyl peptidase III inhibitor、PAM inhibitor、inhibitor of tripeptidyl peptidase II、acylaminoacyl peptidase inhibitor
MPGPLF	Antiamnestic、ACE inhibitor、antithrombotic、regulating、antioxidative、inhibitor、chemotactic、hypotensive、dipeptidyl peptidase IV inhibitor、PAM inhibitor、xaa-pro inhibitor、lactocepin inhibitor、neprilysin inhibitor
FPAPFPASG ARLC	ACE inhibitor、antioxidative、dipeptidyl peptidase IV inhibitor、dipeptidyl peptidase III inhibitor 、ACE2 inhibitor、inhibitor of tripeptidyl peptidase II、neprilysin inhibitor、alanine carboxypeptidase inhibitor、peptidylprolyl isomerase isnhibitor
SHGGFF	ACE inhibitor、dipeptidyl peptidase IV inhibitor、dipeptidyl peptidase III inhibitor 、inhibitor of tripeptidyl peptidase II、pseudolysin inhibitor、neprilysin inhibitor、acylaminoacyl peptidase inhibitor、inhibitor of cytosol alanyl aminopeptidase
GDPNWF	ACE inhibitor、dipeptidyl peptidase IV inhibitor、neprilysin inhibitor
DGGGWW	ACE inhibitor、antioxidative、anti inflammatory、dipeptidyl peptidase IV inhibitor、inhibitor of tripeptidyl peptidase II、neprilysin inhibitor
FGPPPPP	Antiamnestic、ACE inhibitor、antithrombotic、regulating、antioxidative、hypotensive、dipeptidyl peptidase IV inhibitor、alpha-glucosidase inhibitor、neprilysin inhibitor
PGIPPPF	alpha-amylase inhibitor、antiamnestic、ACE inhibitor、antithrombotic、regulating、anti inflammatory、inhibitor、dipeptidyl peptidase IV inhibitor、alpha-glucosidase inhibitor、dipeptidyl peptidase III inhibitor、ACE2 inhibitor、PAM inhibitor
FDPFLR	ACE inhibitor、dipeptidyl peptidase IV inhibitor、alpha-glucosidase inhibitor、dipeptidyl peptidase III inhibitor、renin inhibitor、ACE2 inhibitor、hypouricemic
GDPNWFMK	ACE inhibitor、dipeptidyl peptidase IV inhibitor、dipeptidyl peptidase III inhibitor、neprilysin inhibitor
GPAPPPPLP	Antiamnestic、ACE inhibitor、antithrombotic、regulating、antioxidative、inhibitor、hypotensive、dipeptidyl peptidase IV inhibitor、alpha-glucosidase inhibitor、xaa-pro inhibitor、lactocepin inhibitor、inhibitor of tripeptidyl peptidase II、neprilysin inhibitor、peptidylprolyl isomerase isnhibitor

IDGFFP	ACE inhibitor、dipeptidyl peptidase IV inhibitor、dipeptidyl peptidase III inhibitor、inhibitor of tripeptidyl peptidase II、pseudolysin inhibitor、acylaminoacyl peptidase inhibitor
WPGPGG	Antiamnestic、ACE inhibitor、antithrombotic、regulating、antioxidative、inhibitor、chemotactic、hypotensive、dipeptidyl peptidase IV inhibitor、PAM inhibitor、neprilysin inhibitor
WGPPGG	Antiamnestic、ACE inhibitor、antithrombotic、regulating、antioxidative、hypotensive、dipeptidyl peptidase IV inhibitor、alpha-glucosidase inhibitor、renin inhibitor、PAM inhibitor、neprilysin inhibitor
WCQHYW	ACE inhibitor、anti inflammatory、dipeptidyl peptidase IV inhibitor
FGGGGF	ACE inhibitor、dipeptidyl peptidase IV inhibitor、dipeptidyl peptidase III inhibitor 、inhibitor of tripeptidyl peptidase II、alkaline phosphatase inhibitor、neprilysin inhibitor、acylaminoacyl peptidase inhibitor、inhibitor of cytosol alanyl aminopeptidase
FPKMIF	ACE inhibitor、antioxidative、dipeptidyl peptidase IV inhibitor
PGMNPF	Antiamnestic、ACE inhibitor、antithrombotic、regulating、dipeptidyl peptidase IV inhibitor、dipeptidyl peptidase III inhibitor、ACE2 inhibitor、PAM inhibitor
FFSPGVW	Antiamnestic、ACE inhibitor、antithrombotic、regulating、antioxidative、hypotensive、dipeptidyl peptidase IV inhibitor、alpha-glucosidase inhibitor、PAM inhibitor、pseudolysin inhibitor、hypouricemic
PGGGPF	Antiamnestic、ACE inhibitor、antithrombotic、regulating、antioxidative、hypotensive、dipeptidyl peptidase IV inhibitor、dipeptidyl peptidase III inhibitor 、ACE2 inhibitor、PAM inhibitor、neprilysin inhibitor
FPDFLR	ACE inhibitor、dipeptidyl peptidase IV inhibitor、alpha-glucosidase inhibitor、dipeptidyl peptidase III inhibitor、renin inhibitor、glutamate carboxypeptidase II inhibitor、hypouricemic
PPPGPPPPPG PPPPPG	Antiamnestic、ACE inhibitor、antithrombotic、regulating、antioxidative、inhibitor、chemotactic、hypotensive、dipeptidyl peptidase IV inhibitor、alpha-glucosidase inhibitor、PAM inhibitor、neprilysin inhibitor
WTQRFF	ACE inhibitor、dipeptidyl peptidase IV inhibitor、dipeptidyl peptidase III inhibitor、Leucyltransferase inhibitor、pseudolysin inhibitor
FSWSAF	ACE inhibitor、dipeptidyl peptidase IV inhibitor、alpha-glucosidase inhibitor、pancreatic lipase inhibitor、inhibitor of tripeptidyl peptidase II
DPNWFPK	ACE inhibitor、antioxidative、dipeptidyl peptidase IV inhibitor
DGGAWW	ACE inhibitor、antioxidative、inhibitor、dipeptidyl peptidase IV inhibitor、neprilysin inhibitor、alanine carboxypeptidase inhibitor、inhibitor of cytosol alanyl aminopeptidase
PGGPGF	Antiamnestic、ACE inhibitor、antithrombotic、regulating、antioxidative、hypotensive、dipeptidyl peptidase IV inhibitor、dipeptidyl peptidase III inhibitor、PAM inhibitor、inhibitor of tripeptidyl peptidase II、neprilysin inhibitor、acylaminoacyl peptidase inhibitor
GWNIPMGL	ACE inhibitor、antioxidative、dipeptidyl peptidase IV inhibitor、inhibitor of tripeptidyl peptidase II
GPPPAPPLP	Antiamnestic、ACE inhibitor、antithrombotic、regulating、antioxidative、inhibitor、hypotensive、dipeptidyl peptidase IV inhibitor、alpha-glucosidase inhibitor、xaa-pro inhibitor、lactocepin inhibitor、inhibitor of tripeptidyl peptidase II、neprilysin inhibitor、peptidylprolyl isomerase isnhibitor
MIKPFF	ACE inhibitor、antioxidative、dipeptidyl peptidase IV inhibitor、dipeptidyl peptidase III inhibitor 、ACE2 inhibitor、pseudolysin inhibitor
AGGPFP	Antiamnestic、ACE inhibitor、antithrombotic、regulating、antioxidative、hypotensive、dipeptidyl peptidase IV inhibitor、dipeptidyl peptidase III inhibitor、ACE2 inhibitor、neprilysin inhibitor

WTGSFF	ACE inhibitor、dipeptidyl peptidase IV inhibitor、renin inhibitor、pseudolysin inhibitor
SFPGSF	Antiamnestic、ACE inhibitor、antithrombotic、regulating、dipeptidyl peptidase IV inhibitor renin inhibitor、PAM inhibitor
GPAGMPGFP	Antiamnestic、ACE inhibitor、antithrombotic、regulating、antioxidative、hypotensive、dipeptidyl peptidase IV inhibitor、dipeptidyl peptidase III inhibitor 、PAM inhibitor、inhibitor of tripeptidyl peptidase II、neprilysin inhibitor、acylaminoacyl peptidase inhibitor

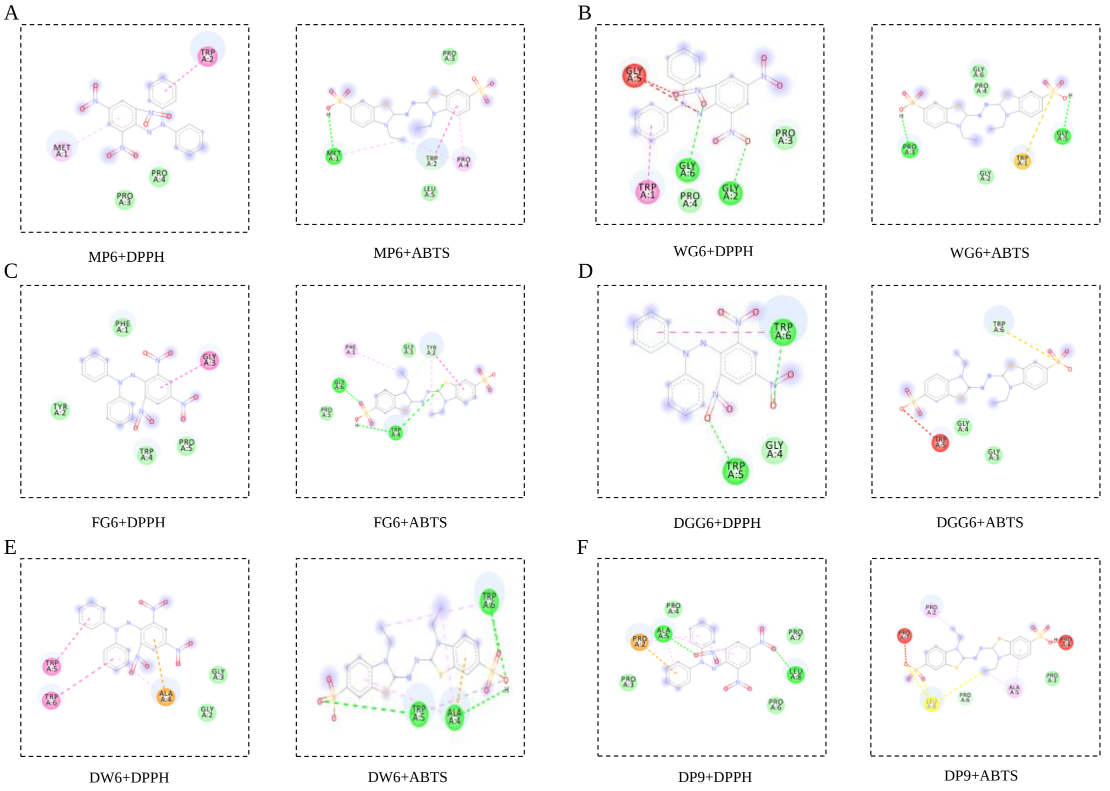

Supplementary Figure S1 Two-dimensional docking diagram of antioxidant peptides with DPPH/ABTS.

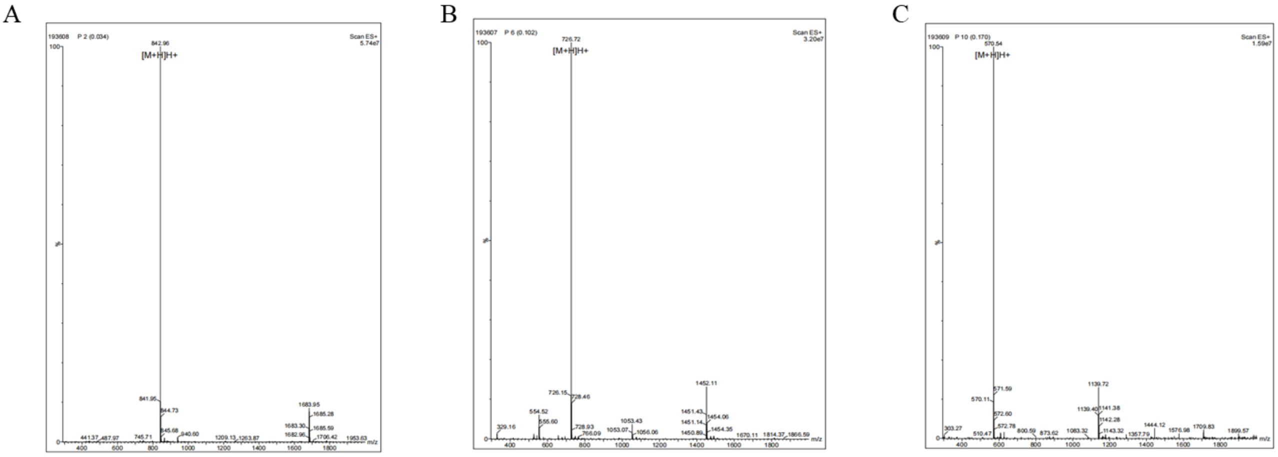

Supplementary Figure S2 Mass spectrum of antioxidant peptides. A: FYGWPG ; B: GPPPAPPLP; C: WGPPGG;
